# Understanding the adult and adolescent patient experience with cyclic vomiting syndrome: a concept elicitation study

**DOI:** 10.1186/s12876-025-03595-7

**Published:** 2025-02-17

**Authors:** Katja Karrento, Melody Wu, Danielle Rodriguez, Karin S. Coyne, Muna J. Tahir, Camilla A. Richmond, Yaozhu J. Chen, James Williams, Thangam Venkatesan

**Affiliations:** 1 Medical College of Wisconsin, Milwaukee, WI USA; 2 Takeda Development Center Americas, Inc., 500 Kendall Street, Cambridge, MA 02142 USA; 3 Evidera, Bethesda, MD USA; 4 The Ohio State University College of Medicine, Columbus, OH USA

**Keywords:** Cyclic vomiting syndrome, Qualitative research, Concept elicitation interviews, Adults, Adolescents, Caregivers

## Abstract

**Background:**

Cyclic vomiting syndrome (CVS) is a phasic disorder of gut–brain interaction characterized by episodes of severe nausea and vomiting. In-depth qualitative research on phase-specific CVS symptoms and impacts is lacking. The study objectives were to explore the experience of patients with CVS in the United States and to identify CVS symptoms and impacts on adults, adolescents, and caregivers.

**Methods:**

Qualitative, cross-sectional, semi-structured concept elicitation interviews were conducted with adults and adolescents with CVS and with adolescents’ caregivers. Adolescents either participated alone or in a dyad format with their caregiver. Interview data were analyzed using an open coding approach.

**Results:**

Concept elicitation interviews were conducted with 13 adults (mean age 45.3 years [standard deviation (SD) 13.1]) and 15 adolescents (mean age 14.6 years [SD 1.8]). The most frequently reported prodrome phase symptoms were nausea (*n* = 12, 92.3%), anxiety (*n* = 10, 76.9%), and abdominal pain (*n* = 9, 69.2%) in adults, and nausea (*n* = 15, 100%), abdominal pain (*n* = 11, 73.3%), and headache (*n* = 11, 73.3%) in adolescents. All adults reported nausea, tiredness, and dry heaves in the emetic phase, and 12 (92.3%) reported vomiting and retching. The remaining patient said they no longer vomited due to abortive medications. All adolescents reported nausea and vomiting in the emetic phase; other common emetic phase symptoms were abdominal pain (*n* = 14, 93.3%), dehydration (*n* = 13, 86.7%), and tiredness (*n* = 13, 86.7%). The leading most bothersome impact reported by adults was anxiety associated with impending vomiting (*n* = 5, 38.5%). Among adolescents, the leading most bothersome impact was on school (*n* = 7/13 asked, 53.8%), and among their caregivers, it was seeing their child suffer (*n* = 6/11 asked, 54.5%).

**Conclusions:**

Patients with CVS experience considerable gastrointestinal and extra-intestinal symptoms. CVS impacts the activities of daily life of patients and their caregivers, with patients reporting negative effects of CVS on their emotional status and their ability to maintain a normal school or work routine.

**Supplementary Information:**

The online version contains supplementary material available at 10.1186/s12876-025-03595-7.

## Introduction

Cyclic vomiting syndrome (CVS) is a chronic disorder of gut–brain interaction (DGBI) that presents as recurrent episodes of severe nausea, vomiting, retching, and abdominal pain [1]. Initially described in children of pre- or early-school age [2], CVS has a reported pediatric prevalence of 1.1–3.4% in the United States [3–5]. Although CVS in children may resolve or evolve into migraine by early adolescence [2], adults also experience CVS, with an estimated prevalence of 2% in the United States [6] and 1.2% worldwide [7].

CVS is generally characterized by four phases: (a) the “prodrome phase,” where patients sense the onset of an episode and experience symptoms such as panic, lethargy, nausea, and abdominal pain; (b) the “emetic phase,” where patients experience persistent and intense nausea, vomiting, and retching that last for hours to days; (c) the “recovery phase,” where CVS symptoms subside and energy and appetite return; and (d) the “well” or “inter-episodic phase,” where patients return to their baseline health [8]. Current understanding of CVS pathophysiology remains limited, and no biomarkers exist to support diagnosis or guide management [9]. In the absence of licensed curative therapies, treatment is complex and requires expertise as well as an understanding of individual factors that need to be targeted therapeutically [10].

CVS episodes are often stereotypical for an individual patient; however, the onset, duration, and severity of symptoms can vary considerably between individuals (for example, some individuals may experience prodromes lasting much longer than the typical 1 h), and symptomatology may change over time [11, 12]. The intensity, length, and unpredictability of episodes negatively impact patients and their caregivers; these impacts include reduced health-related quality of life, work and school absenteeism, short-term disability, increased rates of divorce, and high healthcare utilization and costs [1, 13, 14]. Given the episodic nature of the condition, the true impact of CVS is likely best captured by patients themselves, rather than outcome surveys assessing a brief period.

For other symptomatic gastrointestinal conditions such as gastroparesis, U.S. Food and Drug Administration (FDA) guidance stresses the need for well-defined and reliable patient-reported outcome (PRO) tools that capture clinically important signs and symptoms [15]. Standard PROs are difficult to apply to an episodic illness like CVS [15], and no CVS-specific PRO covering relevant symptom concepts currently exists. Meanwhile, FDA guidance on patient-focused drug development posits that direct input from target populations can provide insight into meaningful concepts that may eventually inform the content of PROs [16]. In contributing to a comprehensive understanding of the unique perspectives of patients with CVS, qualitative methods such as concept elicitation interviews are fundamental to the development of CVS-specific PRO measures [17].

To date, published qualitative research on the impact of phase-specific CVS-related symptoms is limited. It is important to qualitatively assess symptoms and their impacts from a patient perspective to holistically understand the disease experience and to support patient-centered care for different age groups. This study used concept elicitation methods to explore patient and caregiver experiences with CVS and to identify the impact of CVS-specific symptoms on adults, adolescents, and caregivers of adolescents in the United States.

## Methods

A qualitative concept elicitation interview study was conducted among adults with CVS as well as adolescents with CVS and their caregivers. Adult patients were recruited from the tertiary care CVS program at Froedtert Hospital (Wisconsin), whereas adolescent patients and their adult caregivers were recruited from Children’s Wisconsin hospital. Patients were eligible if they had been diagnosed with CVS (based on Rome IV criteria [18]) ≥ 1 year ago, experienced ≥ 2 stereotypic episodes of nausea and vomiting over the last 6 months, and were 18–65 years (adults) or 12–17 years (adolescents) of age. Exclusion criteria were CVS prodrome phase of > 4 h duration before CVS emetic events, cannabis use > 3 days per week, a history of cannabinoid hyperemesis syndrome, chronic opiate use in the last three months, and a history of nausea or vomiting due to other medical conditions or treatments. The sample size was estimated based on the study team’s experience and confirmed based on reaching concept saturation in the interviews. Saturation was reached when no substantially new themes, concept descriptions, or terms were introduced in additional interviews [19, 20].

### Interviews

Potential study participants were identified from site patient databases and outpatient clinics. After obtaining written informed consent and/or assent, the site investigator confirmed the participant’s eligibility. Concept elicitation interviews were conducted remotely using a semi-structured interview guide developed for the study (see Additional File 1). Adolescents could participate in a dyad interview with their caregiver or separately, depending on the adolescent’s preference. All interviews were conducted in English and lasted approximately 90 min. The purpose of the interviews was to gain insights into participants’ CVS experiences, focusing on their symptoms and on how living with CVS symptoms affected their lives. After the interviews, sociodemographic information was collected, and site staff also completed a clinical questionnaire to document CVS history, comorbidities, and medication use. Descriptive statistics were used to summarize participant sociodemographic and clinical data.

### Data processing and analysis

Interviews were audio-recorded, transcribed, and cleaned to remove personal identifiable information. Cleaned transcripts were divided into groups and analyzed via an open coding approach using ATLAS.ti 9.0 (adults) or 22.0 (adolescents) © by ATLAS.ti Scientific Software Development GmbH, Berlin. Coders all received training to ensure that they understood the meaning and purpose of each code and to ensure consistency in transcript coding. Coded transcripts were reviewed by a senior researcher, and analysis was only finalized after coding reconciliation. Qualitative coding of data and concepts played a key role in determining whether saturation of major CVS symptom concepts was reached by the final transcript group.

### Ethics statement

The study was conducted in accordance with the International Council for Harmonisation of Technical Requirements for Pharmaceuticals for Human Use for Good Clinical Practices (ICH E6 [R2]), the Declaration of Helsinki concerning medical research in humans, and appropriate regulatory requirements.

## Results

Overall, 13 adults and 15 adolescents with CVS participated in semi-structured concept elicitation interviews; 14 adolescents participated in a dyad interview with their caregiver, and one adolescent and their caregiver were interviewed separately back to back. Most questions were answered by adolescents, although sometimes responses were provided by caregivers with close involvement in their child’s life and care.

### Demographic and clinical characteristics

#### Adults

Mean (standard deviation [SD]) age of the 13 adult participants was 45.3 (13.1) years. The majority were female (*n* = 9; 69.2%), White (*n* = 10; 76.9%), neither Hispanic nor Latino (*n* = 11; 84.6%), and from the upper Midwest of the United States (Wisconsin and Illinois; *n* = 8; 61.5%). Nearly half the participants reported being employed full-time and all had at least some college education or a technical/vocational degree (Table 1).

**Table 1:** Adult and adolescent patient sociodemographic characteristics

Characteristic	Adults (*n* = 13)	Adolescents (*n* = 15)
Age (years)		
Mean (SD)	45.3 (13.1)	14.6 (1.8)
Median [min, max]	51.0 [26.0, 63.0]	15.0 [12.0, 17.0]
Sex, *n* (% female)	9 (69.2)	8 (53.3)
Racial background, *n* (%)		
Black or African American	2 (15.4)	0
White	10 (76.9)	15 (100)
Asian	1 (7.7)	0
Ethnic background, *n* (%)		
Hispanic or Latino	0	1 (6.7)
Not Hispanic or Latino	11 (84.6)	14 (93.3)
Missing^a^	2 (15.4)	0
Geographic representation, *n* (%)^b^		
Wisconsin	5 (38.5)	8 (53.3)
Illinois	3 (23.1)	2 (13.3)
Ohio	1 (7.7)	0
Pennsylvania	1 (7.7)	0
Rhode Island	1 (7.7)	0
Arkansas	1 (7.7)	0
Georgia	1 (7.7)	0
Florida	0	1 (6.7)
Michigan	0	1 (6.7)
New York	0	1 (6.7)
Alabama	0	1 (6.7)
Minnesota	0	1 (6.7)
Current marital status, *n* (%)		
Single/never married	3 (23.1)	NA
Married or living in marriage-like relationship	7 (53.8)	NA
Widowed/separated/divorced/annulled	1 (7.7)	NA
Missing^a^	2 (15.4)	NA
Current living/domestic situation, *n* (%)		
Living alone	3 (23.1)	NA
Living with a partner or spouse, family, or friends	8 (61.5)	NA
Live with both parents in same home	NA	15 (100)
Missing^a^	2 (15.4)	0
Employment status, *n* (%)		
Work full-time	6 (46.2)	NA
Work part-time	0	NA
Retired	1 (7.7)	NA
Unemployed	2 (15.4)	NA
Disabled	2 (15.4)	NA
Missing^a^	2 (15.4)	NA
Education status, *n* (%)		
Some college	2 (15.4)	NA
College degree	4 (30.8)	NA
Postgraduate degree	3 (23.1)	NA
Technical or vocational degree	2 (15.4)	NA
Missing^a^	2 (15.4)	NA
Current grade, *n* (%)		
7th	NA	3 (20.0)
8th	NA	2 (13.3)
9th	NA	5 (33.3)
10th	NA	1 (6.7)
11th	NA	2 (13.3)
12th	NA	2 (13.3)
Number of days of school missed during a typical CVS episode, *n* (%)		
1 day	NA	1 (6.7)
2 days	NA	5 (33.3)
3 days	NA	2 (13.3)
5 days	NA	1 (6.7)
> 5 days	NA	4 (26.7)
Missing	NA	2 (13.3)

*CVS* cyclic vomiting syndrome, *max *maximum,* min *minimum,* NA* not applicable, *SD* standard deviation

^a^Two adult participants did not return the completed sociodemographic form

^b^Geographic representation data were obtained from the site recruitment log

Mean (SD) time elapsed since CVS diagnosis was 7.9 years (6.1; Table 2). The most common comorbidities were anxiety (*n* = 7; 53.8%) and other DGBIs, e.g., irritable bowel syndrome or functional dyspepsia (*n* = 6; 46.2%). All adults were taking one or more medications to manage their CVS; most (*n* = 11; 84.6%) were taking the rescue medication ondansetron, and about half (*n* = 7; 53.8%) were taking aprepitant and/or sumatriptan. Fewer adults followed prophylactic regimens, including amitriptyline (*n* = 6; 46.2%), topiramate (*n* = 2; 15.4%), and coenzyme Q_10_ (*n* = 2; 15.4%) (Table 2).

**Table 2:** Adult and adolescent patient clinical characteristics

Characteristic	Adults (*n* = 13)	Adolescents (*n* = 15)
Time since CVS diagnosis (years)		
Mean (SD)	7.9 (6.1)	5.0 (2.3)
Median [min, max]	7.5 [0.3, 18.1]	5.8 [1.1, 8.8]
Time in clinician’s practice (years)		
Mean (SD)	5.8 (4.3)	15, 43.9 (33.9)
Median [min, max]	5.0 [0.4, 13.0]	35.0 [1.0, 106.0]
Patient currently receiving CVS treatment, *n* (%)		
Yes	13 (100)	15 (100)
Current CVS medications/treatments, *n* (%)^a^		
Amitriptyline	6 (46.2)	5 (33.3)
Aprepitant	7 (53.8)	11 (73.3)
Benzodiazepine	4 (30.8)	0
Coenzyme Q_10_	2 (15.4)	1 (6.7)
Cyproheptadine	0	1 (6.7)
Diphenhydramine	1 (7.7)	0
Doxylamine/pyridoxine	0	1 (6.7)
Escitalopram	1 (7.7)	0
Fluvoxamine	0	1 (6.7)
Levocarnitine	1 (7.7)	0
Linaclotide	1 (7.7)	0
Lorazepam	0	1 (6.7)
Mirtazapine	2 (15.4)	0
Omeprazole	1 (7.7)	1 (6.7)
Ondansetron	11 (84.6)	15 (100)
Pantoprazole	1 (7.7)	0
Prochlorperazine	1 (7.7)	0
Promethazine	1 (7.7)	1 (6.7)
Propranolol	0	2 (13.3)
Sumatriptan	7 (53.8)	0
Topiramate	2 (15.4)	0
History of comorbid conditions, *n* (%)^a^		
Anxiety	7 (53.8)	8 (53.3)
Asthma	4 (30.8)	3 (20.0)
Depression	4 (30.8)	1 (6.7)
Epilepsy	0	1 (6.7)
Migraine	1 (7.7)	8 (53.3)
Musculoskeletal disorder(s)	0	1 (6.7)
Other disorders of gut–brain interaction^b^	6 (46.2)	12 (80.0)
Other psychological conditions	2 (15.4)	0
Other	6 (46.2)^c^	9 (60.0)^d^
No other health problems	2 (15.4)	0

*CVS* cyclic vomiting syndrome, *max* maximum, *min* minimum, *SD* standard deviation

^a^Not mutually exclusive

^b^Includes disorders such as functional dyspepsia and irritable bowel syndrome

^c^Other comorbid conditions include antiphospholipid antibody syndrome (*n* = 1); benign prostatic hyperplasia and gut neuropathy (*n* = 1); bipolar disorder (*n* = 2); diabetes type 2, insomnia, and hypothyroidism (*n* = 1); diverticulitis, dry eye syndrome, atrophic vaginitis, and presbyopia of both eyes (*n* = 1); hyperlipidemia and diverticulitis of the large intestine (*n* = 1); hypothyroid, lymphoma, diabetes type 2, and Raynaud’s syndrome (*n* = 1); polycystic ovarian syndrome (*n* = 1); pulmonary embolism (*n* = 1); and spinal stenosis and hypothyroidism (*n* = 1)

^d^Other comorbid conditions include attention deficit hyperactivity disorder (*n* = 1), eosinophilic esophagitis (*n* = 1), postural orthostatic tachycardia syndrome (*n* = 1), postural orthostatic tachycardia syndrome and dysautonomia (*n* = 1), postural orthostatic tachycardia syndrome and dizziness (*n* = 1), chronic fatigue (*n* = 1), hypermobile joint syndrome (*n* = 2), orthostatic lightheadedness (*n* = 1), and seizures (*n* = 1)

#### Adolescents

Mean (SD) age of the 15 adolescent participants was 14.6 (1.8) years and about half were female (*n* = 8; 53.3%). All participants were White, and most were neither Hispanic nor Latino (*n* = 14; 93.3%). Although the full adolescent cohort represented participants from seven unique states, about half lived in Wisconsin (*n* = 8; 53.3%) (Table 1). For caregiver sociodemographic characteristics, see Supplementary Table 1, Additional File 2.

Mean (SD) time elapsed since CVS diagnosis was 5.0 (2.3) years (Table 2). The most common comorbidities were other DGBIs (*n* = 12; 80.0%), anxiety (*n* = 8; 53.3%), and migraine (*n* = 8; 53.3%). All adolescents were being treated with ondansetron for CVS management; most (*n* = 11; 73.3%) were being treated with aprepitant, and one-third (*n* = 5; 33.3%) with amitriptyline.

### Triggers of CVS episodes

#### Adults

Almost all adults (*n* = 12/13; 92.3%) reported triggers for vomiting episodes, including stress (*n* = 6/12; 50.0%), specific types of food (e.g., foods high in fat; *n* = 5/12; 41.7%), excitement stemming from positive situations (*n* = 3/12; 25.0%), menstrual cycle (*n* = 3/9; 33.3% of females), and certain smells (*n* = 3/12; 25.0%). They often mentioned multiple triggers, e.g., adult participant ADU04 noted, “*Stress, periods. Smells can be a trigger sometimes. (…) It could be food, perfume, cologne, cleaning products.*” Representative quotes for each section can be found in Supplementary Table 2, Additional File 2. Triggers also included anxiety, alcohol, vacations, and higher temperatures (*n* = 2/12 each; 16.7%), as well as lack of sleep, minty flavors, constipation, and motion sickness (*n* = 1/12 each; 8.3%).

#### Adolescents

Almost all adolescents (*n* = 13/15; 86.7%) reported triggers for vomiting episodes, including stress (*n* = 9/13; 69.2%), getting a cold/flu (*n* = 4/13; 30.8%), specific foods (e.g., dairy; *n* = 3/13; 23.1%), traveling/car rides (*n* = 3/13; 23.1%), and certain smells (*n* = 3/13; 23.1%). Other triggers included exertion, higher temperatures, and lack of sleep (*n* = 2/13 each; 15.4%), menstrual cycle (*n* = 1/8; 12.5% of females), as well as fluoride, light, and skipping a meal (*n* = 1/13 each; 7.7%).

### Frequency and timing of CVS episodes

#### Adults

All 13 adults were asked about the number of CVS attacks experienced in the past year. One participant (7.7%) reported experiencing < 4 episodes, six participants (46.2%) reported 4–7 episodes, and six participants (46.2%) reported ≥ 8 episodes. When asked if there was a particular time of year or season when they tended to experience vomiting episodes, five adults (38.5%) attributed CVS episodes to changes in weather—either cool/cold temperatures in the winter or warm/hot temperatures in the summer. Adult participant ADU12 associated CVS episodes with their holidays by noting, *“I tend to have more attacks during the holiday season (…) I guess it's stress-related because it doesn't necessarily have to be negative stress, it could be positive stress as well that'll trigger.”*

The eight adults who did not report a particular seasonality for their CVS episodes were asked how often they experienced episodes in a typical month. Three (*n* = 3/8; 37.5%) noted that the frequency of their CVS episodes per month varied too much to provide an accurate answer. The responses of the remaining five varied in terms of how often episodes occurred, ranging from four episodes per month to less than one per month or every 3–4 months.

All participants were asked if they experienced episodes at certain times. One participant noted that their CVS episodes were related to their menstrual cycle. At a more granular level, five participants (38.5%) stated that their episodes occurred at certain times of the week, either typically during the work week or over the weekend.

#### Adolescents

Among the 15 adolescents, three (20%) reported experiencing < 4 episodes, two (13.3%) reported 4–7 episodes, and ten (66.7%) reported ≥ 8 episodes. When asked if there was a particular time of year or season when CVS episodes occurred, most adolescents (*n* = 12; 80.0%) said “yes” and five of them (*n* = 5/12; 41.7%) attributed an increase of episodes to the school year because of stress or exposure to illness. Adolescent participant ADO09 said, “*I mean I still have (…) the CVS episodes in the summertime, but in the school year, it's just because of like the stress and anxiety that I have with like my school (…).”* Spring and summer, specific months, “flu season,” and major holidays were also reported to be associated with CVS episodes by adolescents or their caregivers.

Furthermore, of the 12 adolescents who reported a particular time of year when they experienced more CVS episodes, eight (*n* = 8/12; 66.7%) specifically mentioned increased frequency or duration of episodes during such times, ranging from daily episodes (*n* = 1/12; 12.5%) to episodes occurring two to three times per week (*n* = 1/12; 12.5%). One adolescent reported that the episodes occurred “three times more often” during the school year, and another said that the episode could last an extra four days.

All adolescents were asked how often CVS episodes occurred in a typical month. Although two adolescents (13.3%) noted that the frequency of CVS episodes per month varied too much to provide an accurate answer, most (*n* = 9/15; 60.0%) indicated experiencing up to two episodes per month. The remaining participants reported highly variable CVS episode frequencies, ranging from four to 15 episodes per month. In addition, the separately interviewed caregiver reported that their adolescent experienced menstrual cycle–related episodes. On a weekly level, five participants (33.3%) reported episodes occurring at the beginning of the week, including Sunday, or on weekdays in general.

Finally, of the 12 adolescents who indicated that episodes occurred at certain times of the day, the majority (*n* = 8/12; 66.7%) experienced episodes most often in the morning, whereas others reported experiencing episodes at midday/afternoon or evening (*n* = 2/12 each; 16.7%). The adult cohort was not asked about episode occurrence at certain times of the day.

### CVS symptoms during the prodrome phase

#### Adults

**Fig. 1:**
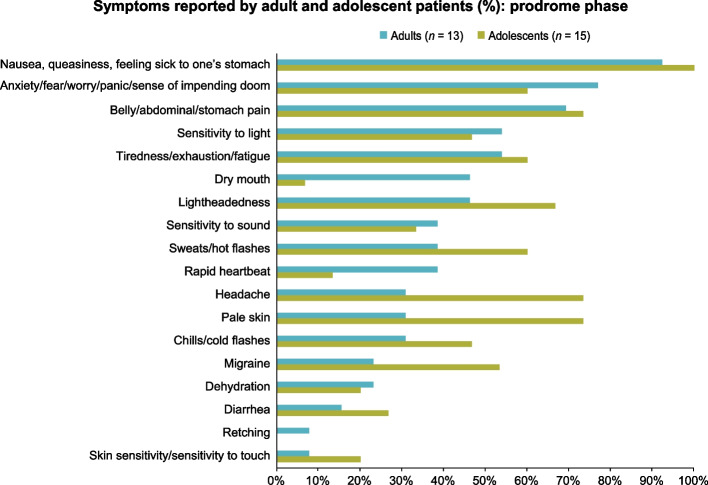
Symptoms reported by adult and adolescent patients (%): prodrome phase

Among adults, saturation of symptom concepts related to the prodrome phase were reached by the third and final transcript group (see Supplementary Table 3, Additional File 2). The most commonly reported symptoms were nausea, queasiness, or feeling sick to one’s stomach (*n* = 12; 92.3%); anxiety, fear, worry, panic, or a sense of impending doom (*n* = 10; 76.9%); belly, abdominal, or stomach pain (*n* = 9; 69.2%); tiredness, exhaustion, or fatigue (*n* = 7; 53.8%); and sensitivity to light (*n* = 7; 53.8%). Other commonly reported symptoms are listed in Fig. 1.

Most adults indicated that their prodrome symptoms persisted for < 1 h. Five adults (38.4%) endorsed < 1 h only, two (15.4%) endorsed < 1 h or 1–2 h, one each (7.7%) endorsed 1–2 h or 3–4 h, and two (15.4%) endorsed 2–3 h. Although participants were required to have a prodrome phase of ≤ 4 h for study inclusion, two adults ultimately endorsed a prodrome duration of > 4 h (15.4%).

#### Adolescents

Among adolescents, saturation of prodrome symptom concepts was reached by the final transcript group (see Supplementary Table 3, Additional File 2). The most prevalent prodrome symptoms among adolescents were nausea (*n* = 15; 100%), headache (*n* = 11; 73.3%), abdominal pain (*n* = 11; 73.3%), pale skin (*n* = 11; 73.3%), and lightheadedness (*n* = 10; 66.7%). Other commonly reported symptoms are listed in Fig. 1.

The prodrome phase lasted < 1 h for six participants (40.0%), whereas another six (40.0%) reported the prodrome phase lasting 1–4 h. Despite study inclusion criteria requiring a prodrome phase of ≤ 4 h, one adolescent reported the prodrome phase lasting all day. Two adolescents (13.3%) remarked that prodrome duration varied too much to be able to answer this question.

### CVS symptoms during the emetic phase

#### Adults

**Fig. 2:**
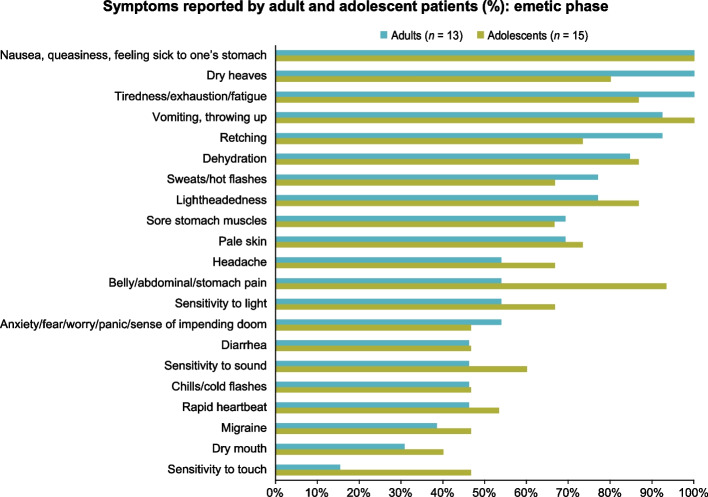
Symptoms reported by adult and adolescent patients (%): emetic phase

Among adults, most symptom concepts related to the emetic phase were first endorsed within the first transcript group (see Supplementary Table 4, Additional File 2). Commonly endorsed emetic phase symptoms were nausea, fatigue, and dry heaves (*n* = 13 each; 100%); vomiting (*n* = 12; 92.3%); and retching (*n* = 12; 92.3%). One participant reported no longer vomiting due to strict adherence to abortive medications. Other symptoms are presented in Fig. 2.

When asked to compare the concepts of vomiting and retching, all participants indicated differences between the two symptoms. Participants noted that vomiting reflected a more productive expunging of stomach contents, whereas retching was associated with the absence of gastric content and with bile and pain. Nonetheless, most (*n* = 11; 84.6%) noted that vomiting and retching were often experienced together.

Almost half the participants (*n* = 6; 46.2%) indicated that the emetic phase lasted for < 1 day; the remaining participants indicated that it ranged anywhere from 1–2 days to 5–6 days.

Of all CVS symptoms, regardless of phase, most adults cited nausea or vomiting as the most bothersome (*n* = 5 each; 38.5%). Two adults (15.4%) indicated that abdominal pain was their most bothersome symptom, and one (7.7%) reported anxiety, fear, worry, panic, or a sense of impending doom.

#### Adolescents

Most emetic phase symptoms among adolescents were first endorsed within the first transcript group (see Supplementary Table 4, Additional File 2). Commonly endorsed emetic phase symptoms were nausea and vomiting (*n* = 15 each; 100%), abdominal pain (*n* = 14; 93.3%), and dehydration, fatigue, and lightheadedness (*n* = 13 each; 86.7%). Adolescent participant ADO01 reported: *“I usually have nausea and if I'm vomiting, I'll usually have like stomach pains. Another thing I usually feel like anxious and upset, obviously.”* Additional symptoms are summarized in Fig. 2.

All 15 adolescents indicated that vomiting and retching were distinct in that vomiting reflected expulsion of stomach contents, whereas retching yielded nothing or only bile/acid. Four adolescents (26.7%), although acknowledging the difference between vomiting and retching, described vomiting and retching as a physically similar action.

Duration of the emetic phase varied greatly among adolescents. Five adolescents (33.3%) reported a duration of up to 2 h, three adolescents and a caregiver (adolescent did not answer) (*n* = 4; 26.7%) reported a duration of 6–12 h, and the rest reported a duration of a few days (*n* = 3; 20.0%) to weeks (*n* = 3; 20.0%).

Most adolescents reported nausea (*n* = 6; 40%), vomiting (*n* = 5; 33.3%), and/or abdominal pain (*n* = 4; 26.7%) as their most bothersome symptoms, regardless of CVS phase. Fewer than 25% reported anxiety, fear, worry, panic, or a sense of impending doom; migraine or headache; and flushing of skin (ears) as most bothersome. The separately interviewed caregiver indicated nausea and vomiting as their adolescent’s most bothersome symptom, whereas their adolescent mentioned anxiety.

The most difficult symptoms for caregivers to manage included vomiting (*n* = 7; 46.7%), nausea (*n* = 4; 26.7%), abdominal pain (*n* = 4; 26.7%), and headaches or migraine (*n* = 2; 13.3%). Some caregivers could not decide on only one symptom as most difficult to manage. Caregiver CG01 stated* “(…) I'm really split between the pain and the vomiting. Vomiting is just awful and it's hard to move her and whatever, but the pain is the one that kills my heart because you can't stop it.”* Anxiety, fear, worry, panic, or a sense of impending doom; being “out of it”; and throat pain were mentioned once each as the most difficult symptom for caregivers to manage.

### Impact of CVS on daily life

#### Adults

All participants were asked how their life was impacted by CVS, and what their most bothersome impacts were. Five adults (38.5%) identified feelings of anxiety related to an imminent vomiting episode as most bothersome, whereas two (15.4%) said it was having to miss work so often. Other most bothersome impacts affected daily activities, social life, and family relationships (for all reported impacts, see Supplementary Table 5, Additional File 2).

#### Adolescents

The most bothersome (not mutually exclusive) impacts for adolescents were the impact on school (*n* = 7/13; 53.8%) and missing out on sports or other social activities (*n* = 4/13; 30.8% each). Other most bothersome impacts reported by one participant each were impacts on sleep, emotional impacts in general, anxiety specifically, and friend relationship impacts.

In a discussion of impacts that were most difficult for caregivers or their family to manage (*n* = 11/15), six caregivers (*n* = 6/11; 54.5%) reported the emotional impact brought on by seeing their child having to go through CVS. Three caregivers (*n* = 3/11; 27.3%) reported the impact related to their child’s schooling, and two (*n* = 2/11; 18.2%) mentioned the impact on their or their family’s social activities. Other most difficult impacts reported by one caregiver each included those on sports, friends/social activities, the adolescent’s future, caregiver’s work, daily planning, and travel planning. All reported impacts from CVS on adolescents and their caregivers are presented in Supplementary Table 5, Additional File 2.

## Discussion

This cross-sectional study used concept elicitation methods to explore patients’ experiences with CVS. To our knowledge, this is the first study to qualitatively explore the symptomatic CVS experience and its impact on health-related quality of life and activities of daily living in adults, adolescents, and caregivers. Although all participants were treated for their CVS, they continued to experience symptoms and associated negative impact. The most commonly experienced gastrointestinal and extra-intestinal CVS symptoms during the prodrome and emetic phases were broadly similar between adults and adolescents. Within both cohorts, prodrome and emetic phase symptoms overlapped; notable exceptions were vomiting and retching, which were, as anticipated, reported only during the emetic phase. The overlapping, disabling symptoms across the prodrome and emetic phases support the practice of therapeutic interventions as early as possible during the prodrome. It is also important to note that CVS episodes varied between individuals in symptoms and duration.

The most common prodrome phase symptoms, reported by over two-thirds of both adults and adolescents, were nausea and abdominal pain. Anxiety was reported by over two-thirds of adults; headache, pale skin, and lightheadedness were reported by over two-thirds of adolescents. The most common emetic phase symptoms, reported by both adults and adolescents, were nausea, dry heaves, vomiting, and retching. Other commonly reported emetic phase symptoms included fatigue, dehydration, hot flashes, lightheadedness, pale skin, and sore stomach muscles.

In adolescents, but not in adults, migraine was among the top ten symptoms during the prodrome phase and headache was among the top ten symptoms during the emetic phase. This mirrored both the higher prevalence of migraine history in adolescents (*n* = 8; 53.3% vs. *n* = 1; 7.7% in adults) and similar results in other studies [21, 22]. For both adults and adolescents, the most common prodrome and emetic phase symptoms were also the most bothersome.

Migraine comorbidity and a family history of migraine are commonly associated with CVS [1, 2], and studies show that approximately 40–70% of pediatric patients who outgrow their CVS develop migraine headaches in adulthood [23–25]. In line with this, a recent longitudinal study of pediatric patients with CVS in Italy identified resolution of vomiting and development or recurrence of migraine as one of four disease outcomes [26]. Our findings add to this data and may help identify important efficacy targets for future research.

Although some adults and adolescents reported their emetic phase as lasting less than a day, other adults reported 5- to 6-day emetic phases and other adolescents reported weeks-long emetic phases. Existing consensus-driven diagnostic guidelines define CVS episodes as lasting either less than a week or up to 10 days, but do not define the end of an CVS episode [27, 28]. Rather than experiencing atypically long episodes, it is possible that participants may have instead experienced back-to-back episodes or coalescent CVS [29]. Indeed, recent research suggests that a significant proportion of pediatric patients with CVS may develop chronic rather than episodic symptoms of autonomic nervous system dysfunction. Such patients are more likely to miss school due to chronicity of symptoms and may experience greater impacts on their health-related quality of life [30]. The burden of CVS on younger patients and their caregivers may thus be higher than previously reported.

All participants described substantial impact of CVS on their daily lives, with anxiety in particular playing a big part in the disease experience of adults. A recent 15-year retrospective study of CVS patients at the Medical College of Wisconsin found that 80.7% of patients did not achieve episode resolution over a mean follow-up of 47.4 months and that 4% of patients died during the study period. Although causation remained unproven, the authors suspected that some of these deaths were due to overdose; the authors mentioned that, in their clinical experience, many patients with CVS are accused of drug seeking and lose faith in the healthcare system [31]. This aligns with a 2015 survey that identified perceived lack of knowledge among providers as one of the biggest CVS-related challenges for patients [32].

Most bothersome CVS impacts varied by participant age group, with adults most frequently reporting anxiety and work impacts, and adolescents mostly citing impact on school, social activities, and sports. Anxiety is a prevalent comorbidity among the CVS population, including school-aged children, and studies have identified anxiety symptoms as a predictor of higher impact of the child’s illness on themselves and their families [33, 34]. Caregivers of adolescents indicated that the emotional impact of seeing their child suffer from CVS, and the impact of CVS on their child’s schooling and social activities, were most bothersome to their family. These findings complement reports in existing literature on decreased health-related quality of life, functional disability, increased healthcare utilization and costs, and productivity loss resulting from CVS [13, 14, 34–36].

A strength of this study was the use of a semi-structured interview approach, which offered in-depth insights into patient experiences, beyond those obtained via quantitative studies. Other strengths were the inclusion of caregivers and the opportunity for adolescents and caregivers to participate in dyad interviews, facilitating consolidated capture of broader experiences and, in turn, a deeper understanding of how better to support both parties. While adolescents may not disclose the same information in a dyad versus individual interview, the study team offered adolescent participants the opportunity to interview together with their caregiver in case they would not feel comfortable participating alone. In addition, the interviewed adults, adolescents, and caregivers likely had an in-depth understanding of CVS and CVS-related terminology, having lived with the condition for many years.

Although the clinical sites made every effort to recruit a diverse participant sample, a limitation of this study is the lack of diversity in geographic location, as most participants were recruited from the upper Midwest (where the two sites are located). Although the small sample size may further limit generalizability of our results, concept saturation was reached and the cohort demographics, i.e., primarily female and White, align with current understanding of CVS epidemiology. Because this study relied on retrospective recall of CVS episodes, limited symptom and impact recall and interpretation (e.g., non-migraine headache vs. migraine) may have introduced a possible bias. Social desirability or interviewer biases were likely overcome by interviewer training and the use of a semi-structured interview guide. Finally, given that convenience sampling was used to recruit participants, it may have led to self-selection bias wherein participants differ systematically from other patients with CVS, perhaps in symptom severity or time since disease onset.

## Conclusion

This qualitative study highlights the highly variable symptomatic and humanistic burden of CVS experienced by adults, adolescents, and caregivers of adolescent patients. It underscores the hitherto unmet need for an approved treatment for CVS and may help to define meaningful treatment benefits. Importantly, the diverse CVS prodrome and emetic phase symptoms captured in this study can help inform future CVS-specific PROs.

## Supplementary Information

Additional file 1. Semi-structured interview guides. Semi-structured interview guides for adult participants, adolescent and caregiver dyad participants, and caregiver participants.

Additional file 2. Supplementary tables. Tables on caregiver sociodemographic characteristics, CVS concept elicitation interview quotes, saturation of symptom concepts in the prodrome phase, saturation of symptom concepts in the emetic phase, and CVS impacts.

## Data Availability

The datasets generated and/or analyzed during the current study are not publicly available but are available from the corresponding author on reasonable request.

## Abbreviations

CVS: Cyclic vomiting syndrome
DGBI: Disorder of gut–brain interaction
FDA: Food and Drug Administration
PRO: Patient-reported outcome
SD: Standard deviation
