# Supplementary material for: Understanding the adult and adolescent patient experience with cyclic vomiting syndrome: a concept elicitation study
Source: BMC Gastroenterol. 2025 Feb 17;25:85. doi: 10.1186/s12876-025-03595-7 (PMC11834555; doi:10.1186/s12876-025-03595-7)
Supplement: Supplementary file 1 — Additional file 1. Semi-structured interview guides. Semi-structured interview guides for adult participants, adolescent and caregiver dyad participants, and caregiver participants. [file 12876_2025_3595_MOESM1_ESM.docx]

Contents

[Adult Interview: Semi-Structured Interview Guide 2](#_Toc165552613)

[Introduction 2](#_Toc165552614)

[Background and Consent 2](#_Toc165552615)

[Discussion Rules 3](#_Toc165552616)

[Concept Elicitation 3](#_Toc165552617)

[Experience with Cyclic Vomiting Syndrome 3](#_Toc165552618)

[Symptom Recording Sheet 5](#_Toc165552619)

[Impact of CVS on Daily Life 5](#_Toc165552620)

[Triggers and Treatments 8](#_Toc165552621)

[Wrap-up/Conclusions 8](#_Toc165552622)

[Adolescent Patient and Caregiver Dyad Interview: Semi-structured Interview Guide 9](#_Toc165552623)

[Introduction 9](#_Toc165552624)

[Background 9](#_Toc165552625)

[Discussion Rules 11](#_Toc165552626)

[Concept Elicitation 11](#_Toc165552627)

[Experience with Cyclic Vomiting Syndrome 11](#_Toc165552628)

[Symptom Recording Sheet 15](#_Toc165552629)

[Impact of CVS 16](#_Toc165552630)

[Impact Recording Sheet 17](#_Toc165552631)

[Wrap-up/Conclusions 18](#_Toc165552632)

[Patient Caregiver Only Interview: Semi-structured Interview Guide 19](#_Toc165552633)

[Introduction 19](#_Toc165552634)

[Background 19](#_Toc165552635)

[Discussion Rules 20](#_Toc165552636)

[Concept Elicitation 20](#_Toc165552637)

[Experience with Cyclic Vomiting Syndrome 21](#_Toc165552638)

[Symptom Recording Sheet 23](#_Toc165552639)

[Impact of CVS 25](#_Toc165552640)

[Impact Recording Sheet 26](#_Toc165552641)

[Wrap-up/Conclusions 27](#_Toc165552642)

# Adult Interview: Semi-Structured Interview Guide

**Participant ID: ___________ Participant Initials: _______ Date (MM/DD/YYYY): ____/____/____**

**Instructions for the Interviewer:**

**This discussion guide is meant to help focus the discussion but should not be used as a verbatim script; probes and questions may change slightly depending on participant feedback. Additional unscripted probes to be used to gain further information or clarification may include:**

- Clarification: I don’t quite understand that.
- Expressing understanding: How did you cope with that?
- Justification: What makes you say that?
- Importance: I’m not sure how these two things are linked.
- Extending narrative: Tell me a bit more about that.
- Accuracy: Let’s see if I’ve got that right.

**The interviewer will introduce themselves and explain the study background and interview process. The interview session will be approximately 90 minutes of speaking and writing. The interviewer may adapt the guide to cover the topics in the amount of time allotted for the session.**

Introduction

Background and Consent

Good [**morning/afternoon/evening**]. I am [**interviewer name**], a research professional working with Evidera, a group focusing on patient-centered research. As part of this study, Evidera is planning to interview about 25 patients like you, who have been diagnosed with cyclic vomiting syndrome or CVS. The study is sponsored by Takeda, a pharmaceutical company, who is developing a treatment for symptoms related to CVS.

Thank you for taking the time to participate in this interview. The information that you share with us today will help us better understand the experience of people living with CVS and help us develop a new questionnaire to measure CVS symptoms. Your input is extremely helpful in this endeavor.

The focus of our discussion today will be on the symptoms related to your CVS, including the impact of these symptoms on your day to day life.

**At this time, I would like to confirm that you have completed the electronic consent form.**

**[If no, interviewer to reschedule with the participant]**

**[If yes]** Great. As mentioned in the consent form, the interview will be audio-recorded so that I can capture everything that you say during the interview and review it later. A word-for-word transcript or written documentation of the audio-recording will be prepared. Your privacy will be protected, and the transcript will not include any mention of your name or information that could be used to discover your identity. You will be identified only with an identification (ID) number to protect your confidentiality.

**Is it okay for me to record the conversation today?**

**If yes, continue.**

**If no, restate information that is present on the consent form**: As was discussed on the consent form, your name will not be linked with the recording, transcription, or responses during the interview. Unfortunately, since you do not agree to the recording of this session, you won’t be able to participate in the study. Thank you for your willingness to participate in this study.

Discussion Rules

Before we begin, I’d like to review a few points that will make our discussion more productive.

- There are no right or wrong answers because everyone's experiences are different. Please feel free to share your point of view about how CVS affects you.
- My role here is to ask questions and to listen. I’ll also be summarizing things you’ve said at times to make sure I understand correctly. I’ll ask questions to obtain your feedback, and I’ll move the discussion from one question to the next, to try to keep us on track so we can finish within the allotted time. Some of my questions might seem repetitive or seem like they have obvious answers, but please bear with me and feel free to ask questions or seek clarification at any time.
- Please talk clearly and speak up. We’re audio-recording the session because we don’t want to miss any of your comments and feedback.
- We’ll be talking together on a first-name basis, but later in the report, no names will be included or attached to any of your comments.
- I am not a medical doctor. I am a research professional. My background is in conducting various types of research, so I am not qualified to give out medical advice and I may ask you to clarify anything with which I am not familiar.

**Do you have any questions before we begin?**

**Begin Recorder**: This is participant ID [**insert Participant ID number here]**, and today is [**date**]. I want to confirm that you have reviewed and signed electronically the consent form, you agree to participate in this study, and that you agree to today’s interview being audio-recorded. **Is that correct?**

Concept Elicitation

Experience with Cyclic Vomiting Syndrome

First, I will ask a few questions about your experiences with cyclic vomiting syndrome. I’ll refer to cyclic vomiting syndrome as “CVS” moving forward.

1. How long have you been living with CVS?
2. Do you remember when you were first diagnosed with CVS?
3. Do you recall what issues or symptoms led you to go to the doctor?
4. Were you diagnosed with other issues or conditions before receiving the correct diagnosis? Was it difficult to get the correct diagnosis?

I would like to understand your experience with CVS and how it affects your life.

1. First, can you walk me through what it’s like for you to live with CVS?
2. How often do you tend to have vomiting episodes in a typical month?
   1. [If patients have fewer than 1 episode/monthly] How many vomiting episodes do you think you’ve had over the past year?

People living with CVS can experience a wide variety of different symptoms depending on the individual. As you may know, people tend to experience different phases or cycles with their CVS where sometimes they feel well and other times where they don’t feel well.

1. Is that true for you? [If yes] Can you describe the different phases you go through and how you feel during each phase?
2. How long does each phase usually last for you?
3. What does a typical prodrome phase feel like to you? The prodrome phase is the phase that comes right before a vomiting episode, when you might start to sense that an episode is coming. This phase can last for minutes or hours.
4. How do you know you’ve entered the prodrome phase? What symptoms or feelings do you have?
5. Are these prodrome symptoms or feelings the same each time or can they feel more severe or less severe sometimes? Please explain.
6. How long do these symptoms usually last before vomiting starts?
7. Can you describe what a typical vomiting episode is like for you?
8. How long does it last?
9. How many times will you vomit in a typical episode?
10. Do your vomiting episodes change in severity? In other words, are some episodes of vomiting worse than others?
11. Can you describe a particularly severe vomiting episode that you’ve experienced? What happened and why do you consider that one severe?
12. What about a less severe or mild episode of vomiting – can you describe what a mild episode was like for you?
13. If you could create a complete list of symptoms that you deal with because of CVS, what would be on your list? **[Interviewer: Complete Symptom Recording Table]**
14. Which symptom would you say is the most bothersome of all the symptoms you experience? Please explain why you chose this one.

Symptom Recording Sheet

***Note to interviewer: Please use this table to record the symptoms as stated by participants. Please ensure to record words and terms as used by participants. Probe on any symptoms that they have not already spontaneously discussed by the participant.***

| **Symptoms** | **Spontaneously reported (S) or on probing (P)?** | |
| --- | --- | --- |
|  | **S** | **P** |
| Nausea, queasiness, feeling sick to one’s stomach |  |  |
| Vomiting, throwing up |  |  |
| Dry heaves |  |  |
| Retching |  |  |
| Headache |  |  |
| Migraine |  |  |
| Diarrhea |  |  |
| Pale skin |  |  |
| Belly/abdominal/stomach pain |  |  |
| Sensitivity to light |  |  |
| Sensitivity to sound |  |  |
| Dehydration |  |  |
| Tiredness/exhaustion |  |  |
| Sweating |  |  |
| Rapid heartbeat |  |  |
| Dry mouth |  |  |
| Light headedness |  |  |
| Anxiety |  |  |
| Sense of impending doom |  |  |
| Panic |  |  |
| Fear |  |  |
| Other: |  |  |
| Other: |  |  |
| Other: |  |  |

Impact of CVS on Daily Life

*[Interviewer: For this portion of the guide, please use Table 2 (below) to help document any specific impacts that the participant spontaneously mentions.* ***Do NOT probe on each impact description listed in Table 2****.]*

Now, let’s talk about how CVS affects your daily life. There are four commonly known phases in the full CVS cycle *[Interviewer to describe the four phases illustrated below]*. I’d like to know how CVS and symptoms of CVS impact you at different points in this cycle.

1. How does CVS impact your everyday or daily life? [If yes] In what ways?
2. Would you say that CVS impacts your life when you’re feeling well and in between episodes?
3. How is your life impacted when you start to sense an episode of vomiting is coming on (i.e., impacts during a prodrome phase)?
4. How is your life impacted while you’re experiencing an episode of vomiting?
5. How are you impacted after an episode of vomiting, when you’re recovering from the event?
   1. How long does it typically take for you to recover from the vomiting episode?
6. Does your CVS ever interrupt your sleep or wake you up at night? [If yes] Please explain how CVS can affect your sleep.
   1. When in the CVS cycle (i.e., the well phase, prodrome phase, episode or vomiting phase, recovery phase) is your sleep impacted?
7. Does your CVS prevent you from doing physical activities (e.g., walking, climbing stairs, exercising)? Please explain.
   1. When in the CVS cycle are your physical activities impacted?
8. Does your CVS prevent you from doing daily activities (e.g., household chores, gardening) that you normally do?
   1. When in the CVS cycle are your daily activities impacted? Please explain.
9. Is your work (or school life, if applicable) ever affected by your CVS? Please explain.
   1. When in the CVS cycle is your work or school impacted? Please explain.
10. Does your CVS affect your mood or affect you emotionally? Please explain.
    1. When in the CVS cycle is your mood or emotions impacted? Please explain.
    2. Does your mood change depending on what phase you might be in? Please explain.
11. Does your CVS ever interfere with your social life (e.g., going out for dinner, participating in team sports)? Please explain.
    1. When in the CVS cycle is your mood impacted? Please explain.
12. Does your CVS affect any of your relationships? For example, your relationships with relatives, partner or spouse (if applicable), colleagues, or friends? Please explain.
13. Of all the issues we’ve discussed, which one would you say is the **worst or most bothersome** [probe with impacts mentioned, if needed]? Why?
14. Have you tried treatment for CVS? If yes, what and how much have they helped?

Table 2. CVS Impacts

| Impact | Impact Description | Participant’s Language | Notes |
| --- | --- | --- | --- |
| Sleep (generally)  🞎S 🞎P | 🞎 Ability to fall asleep  🞎 Ability to stay asleep  🞎 Ability to fall back to sleep  🞎 Wake up feeling rested |  |  |
| Physical impacts (generally)  🞎S 🞎P | 🞎 Ability to exercise  🞎 Ability to walk or climb stairs  🞎 Needing to rest  🞎 Other |  |  |
| Daily Activities (generally)  🞎S 🞎P | **Day-to-day activities** 🞎 Chores/housework/gardening  🞎 Daily routine/planning  🞎 Self-care practices  🞎 Other |  |  |
| Work or school (generally)  🞎S 🞎P | **Work or school**  🞎 Daily work/school routine  🞎 Engage in work/school-related activities  🞎 Work/school absenteeism  🞎 Productivity while at work |  |  |
| Emotions or mood (generally)  🞎S 🞎P | **Feelings or mood**  🞎 Depression  🞎 Anxiety  🞎 Panic  🞎 Irritability  🞎 Embarrassment  🞎 Worry  🞎 Stress  🞎 Frustration  🞎 Other |  |  |
| Social life (generally)  🞎S 🞎P | **Social life**  🞎 Social activities  (e.g. going out -cinema, bar or dinner)  🞎 Leisure activities/hobbies  🞎 Sports (e.g. swimming)  🞎 Travel  🞎 Other |  |  |
| Relationships (generally)  🞎S 🞎P | **Relationships**  🞎 Family relationships  🞎 Friend relationships  🞎 Work/school relationships  🞎 Other |  |  |
| Other: specify |  |  |  |

Note: S = Spontaneous; P = Probed

Triggers and Treatments

I’d like to talk about different triggers you may have experienced and different ways you treat your CVS.

1. Do you notice certain triggers for a vomiting episode? [If yes] Please describe.
2. What helps you feel better or cope with the intensity of a typical vomiting episode? Please explain.

Wrap-up/Conclusions

Thank you so much for your time today. The information that you have shared will help us better understand the experiences of people living with CVS and help us develop a new questionnaire to measure your CVS symptoms. Your input is extremely helpful.

**END INTERVIEW HERE**

# Adolescent Patient and Caregiver Dyad Interview: Semi-structured Interview Guide

**Participant ID: ___________ Verification Code: _______ Date (MM/DD/YYYY): ____/____/____**

**Instructions for the Interviewer:**

**The purpose of this interview is to capture the CVS experience of both the adolescent patient and their adult caregiver. Interviews will be conducted as one of two scenarios:**

- **Scenario A:** Adolescents and caregivers are interviewed together. In this case, questions should be addressed to both participants. However, the perspective of the adolescent patient should be the focus of the interview. Therefore, they should be asked the questions first, and then the adult caregiver can supplement the adolescent’s responses with additional information or clarifications. In some instances, questions will be directed only to the caregiver. These specific questions have been labeled as “To the Caregiver.”
- **Scenario B:** Adolescents and caregivers are interviewed separately. In this case, all adolescent‑specific questions from this guide will be applicable. However, those labeled “To the Caregiver,” should not be asked of the adolescent.

**This discussion guide is meant to help focus the discussion but should not be used as a verbatim script; probes and questions may change slightly depending on participant feedback. Additional unscripted probes have been included and should be used to gain further information or clarification. These may include the following:**

Clarification: I don’t quite understand that.

Expressing understanding: How did you cope with that?

Justification: What makes you say that?

Importance: I’m not sure how these two things are linked. Can you please clarify?

Extending narrative: Tell me a bit more about that.

Accuracy: Let’s see if I’ve got that right.

**The interviewer will introduce themselves and explain the study background and interview process. The interview session will be approximately 60-75 minutes of speaking and writing. The interviewer may adapt the guide to cover the topics in the amount of time allotted for the session.**

Introduction

Background

***Note****: For dyad interviews, which include both the adolescent patient and their adult caregiver, read* ***Scenario A*** *text below to the participants. For interviews where adolescent patients are to be interviewed separately from their adult caregiver, read* ***Scenario B*** *text below to the adolescent participant.*

#### Scenario A Text: (Adolescent Patient with Adult Caregiver [Together] Interview)

Good [**morning/afternoon/evening**]. I am [**interviewer name**], a research professional working with Evidera, a group focusing on patient-centered research. Thank you for taking the time to participate in this interview. As part of this study, Evidera is planning to interview about 12 adolescent patients like you, who have been diagnosed with cyclic vomiting syndrome, or CVS, along with their adult caregiver. The study is sponsored by Takeda, a pharmaceutical company, who is developing a treatment for symptoms related to CVS.

The information that you share with us today will help us better understand the experience of people living with CVS and develop a new questionnaire to measure CVS symptoms. Your input is extremely helpful.

The focus of our discussion today will be on the symptoms related to your CVS, including the impact of these symptoms on your day-to-day life. The questions I will be asking today will be directed to you as the adolescent living with CVS. From time to time, I will ask your caregiver a similar or different question to see if they can provide any additional information about your CVS.

#### Scenario B Text: (Adolescent Patient [Only] Interview)

Good [**morning/afternoon/evening**]. I am [**interviewer name**], a research professional working with Evidera, a group focusing on patient-centered research. Thank you for taking the time to participate in this interview. As part of this study, Evidera is planning to interview about 12 adolescent patients like you, who have been diagnosed with cyclic vomiting syndrome, or CVS. Based on your preference, your adult caregiver will be interviewed separately. The study is sponsored by Takeda, a pharmaceutical company, who is developing a treatment for symptoms related to CVS.

The information that you share with us today will help us better understand the experience of people living with CVS and develop a new questionnaire to measure CVS symptoms. Your input is extremely helpful.

The focus of our discussion today will be on the symptoms related to your CVS, including the impact of these symptoms on your day-to-day life.

***Note:*** *Continue with text below with either scenario A or B.*

As mentioned in the [**assent and consent forms/assent form**], we would like to audio-record today’s interview, so that I can capture everything that you say and review it later. A word-for-word transcript or written documentation of the audio-recording will be prepared. Your privacy will be protected, and the transcript will not include any mention of your name, your caregiver’s name, or information that could be used to discover your identity. You will be identified only with an identification (ID) number to protect your confidentiality.

Is it okay for me to record the conversation today?

**If yes, continue.**

**If no, state the following**: That’s alright. I will be taking detailed notes during the interview, and I may ask you to repeat certain responses, so I capture your information accurately.

Discussion Rules

Before we begin, I’d like to review a few points that will make our discussion more productive.

- There are no right or wrong answers because everyone's experiences are different. Please feel free to share your point of view about how CVS affects you.
- **Read only for dyad interviews:** *As an adolescent living with CVS, I’d like to hear from you first when I ask questions. I ask that your caregiver allow you to answer first and then provide additional information as needed. Sometimes I will ask your caregiver questions directly, but I will make that known to you both.*
- My role here is to ask questions and to listen. I’ll also be summarizing things you’ve said at times to make sure I understand correctly. I’ll ask questions to obtain your feedback, and I’ll move the discussion from one question to the next, to try to keep us on track so we can finish within the allotted time. Some of my questions might seem repetitive or seem like they have obvious answers, but please bear with me and feel free to ask questions or seek clarification at any time.
- Please talk clearly and speak up as I don’t want to miss any of your comments and feedback.
- We’ll be talking together on a first-name basis, but later in the report, no names will be included or attached to any of your comments.
- I am not a medical doctor so I am not qualified to give out medical advice, and I may ask you to clarify anything with which I am not familiar.

**Do you have any questions before we begin?**

***Note: Read statement below only if participant(s) gave permission to audio-record the interview.***

**Begin Recorder**: This is participant ID [**insert Participant ID number here]**, and today is [**date**]. I want to confirm that you, the patient, have reviewed and signed the assent form, **[and your caregiver has reviewed and signed the consent form]**, and that you **[both]** agree to participate in this study, and that you **[both]** agree to today’s interview being audio-recorded. Is that correct?

Concept Elicitation

So far, I have referred to your cyclic vomiting syndrome as CVS. Before we get to the interview questions, I’d like to know from you if there is another name you would prefer to call your condition.

**Can you please tell me what it is and how you would like me to refer to it?**

***For dyad interviews***: **Caregiver, can you also let me know how you refer to CVS?**

Experience with Cyclic Vomiting Syndrome

#### Diagnosis History and General Experience of CVS

I’ll refer to cyclic vomiting syndrome as [CVS/participant language] moving forward. First, I will ask a few questions about your experiences with [CVS/participant language].

**Dyad interview only:** If this first set of questions is hard for you to answer we can discuss them with your parent or guardian.

1. How long have you had [CVS/participant language]?
2. Do you remember when you went to the doctor for [CVS/participant language]?
3. Do you remember what issues or symptoms led you to go to the doctor?

**To the Caregiver:**

1. Do you remember when your adolescent was first told by a doctor that they have [CVS/participant language]?
2. Do you recall what issues or symptoms led them to go to the doctor?
3. Was your adolescent told that he/she had other issues or medical conditions before doctors told you that they have [CVS/participant language]? Was it difficult to get the correct diagnosis?
4. Is there any further information to add to what your child described?

#### Experience of CVS Phases in General

As you may know, people experience different phases (or certain periods of time) with their [CVS/PARTICIPANT LANGUAGE] where sometimes they feel well and other times where they don’t feel well.

1. Is that true for you with [CVS/PARTICIPANT LANGUAGE]?
2. [If yes] Can you describe the different phases you go through and how they feel during each phase?
3. How long does each phase usually last for you?
4. What do you call each time period between [CVS/PARTICIPANT LANGUAGE] episodes before you begin to feel a new [CVS/PARTICIPANT LANGUAGE] episode coming on?
5. [If no] Can you describe how you are feeling overall during a [CVS/PARTICIPANT LANGUAGE] episode?

#### Experience of Prodrome Phase

Some people have symptoms that come right before the vomiting phase where they may feel sick and feel a vomiting attack is coming. This phase can last for minutes or hours.

1. Do you experience this? If so, what do you call this phase?
2. How do you define the beginning and the end of a [prodrome/alternative language]? What typically happens to signal the end of a [prodrome/alternative language]?
3. What does a typical [prodrome/alternative language] phase feel like to you?
4. What [prodrome/alternative language] symptoms do you have? [Interviewer to record symptoms on the Symptom Recording Sheet in the **prodrome column**]
5. Do your [prodrome/alternative language] symptoms change with each [prodrome/alternative language] phase that you experience or are they about the same each time? Please explain.
6. If probes are needed, ask about severity, frequency, and duration of prodrome symptoms.
7. How long do these symptoms usually last before the vomiting starts?
8. Do you ever have [prodrome/alternative language] symptoms but then do NOT go on to have a vomiting attack?
9. [If yes] How often does that happen for you?
10. [If yes] Does this happen on its own (i.e., naturally) or do you take medications to prevent the vomiting?

**To the Caregiver:**

1. Is there any further information you wish to add to what your child described?
2. Can you tell when your child is going to have a vomiting attack?
3. Are there specific triggers to a [prodrome/alternate language] phase?

#### Experience of Emetic Phase

1. Can you describe what a typical vomiting phase or attack is like for you?
2. What do you usually call the vomiting phase? How do you define the beginning and the end of a [vomiting attack/alternate language]? What typically happens to signal the end of a [vomiting attack/alternate language]?
3. What symptoms do you experience during a [vomiting attack/alternate language]? [Interviewer to record symptoms on the Symptom Recording Sheet in the **emesis column**]
4. Are these symptoms the same each time you have a [vomiting attack/alternate language] or do you have different symptoms with each episode? Please explain.
5. How long does a typical [vomiting attack/alternate language] last for you?
6. In your mind, is there a difference between retching and vomiting? [If no or patient doesn’t know the term, explain that retching is usually defined as a movement or sound of vomiting but without bringing up any contents, liquid, or solids] How do you prefer to quantify them? [Probe: frequency of vomiting/retching movements, or length of time, or other?]
7. Do you consider dry heaves to be the same as retching? [If different] Please explain.
8. On average, how many times do you vomit (or retch [if considered same as vomiting]) in a typical [vomiting attack/alternate language]?
9. Do your [vomiting attacks/alternate language] change (e.g., in how bad, how often, or for how long they last)? In other words, are some [vomiting attacks/alternate language] worse/more frequent/longer than others or are they the same?

**To the Caregiver:**

1. What is your opinion on the terms retching and vomiting? Do these terms mean the same to you?
2. Is there any further information you wish to add to what your child described?
3. How would you describe a typical [vomiting attack/alternate language] for your child?

#### Frequency and Timing of Episodes with Vomiting

1. How do you define the beginning and end of a CVS episode, which can include more than one phase? What typically happens to signal the end of a CVS episode?
2. How many [CVS/PARTICIPANT LANGUAGE] episodes do you think you’ve had over the past year? ***[******Interviewer, if necessary, clarify that a CVS episode is being discussed using participant’s own language or the following text: When I say episode, I am talking about from when you start to not feel well to being sick until you start to feel well again.]***
3. Is there a certain time of year or season when you tend to experience more episodes compared with others (e.g., during the school year or during the summer)?
4. [If yes] Please explain.
5. In the time of year or season when you experience more episodes, how often do they occur? ***[Interviewer to probe if it is daily, weekly, or less frequently]***

[If no] How often do you tend to have [CVS/PARTICIPANT LANGUAGE]episodes in a typical month?

1. Do you notice that you experience episodes at certain times of the month?
2. [If yes] Please explain. ***[Interviewer to probe if it is earlier, middle, or later in month and why that may be]***
3. What about certain times of the week? For example, are your episodes more likely to occur on weekdays rather than over the weekend?
4. [If yes] Please explain. ***[Interviewer to probe if it is start, middle, or end in the week and why that may be]***
5. Do you tend to have episodes at a certain time of day? For example, are you more likely to experience an episode at night versus in the morning?
6. [If yes] Please explain.

**To the Caregiver:**

1. Is there any further information you wish to add to what your child described?
2. How does the frequency and timing of your child’s [CVS/PARTICIPANT LANGUAGE] episodes impact you and your family?

#### Symptoms Experienced During Prodrome Phase and Emetic Phase

People living with [CVS/PARTICIPANT LANGUAGE] can experience many different symptoms depending on the person. We’ve already discussed some of the different symptoms you experience during different phases, namely during the [prodrome/alternative language] phase and during your [vomiting attacks/alternate language]. We want to be sure to record all symptoms that you have ever experienced during each phase.

I’m going to go through a list of symptoms and ask which ones you have experienced because of your [CVS/PARTICIPANT LANGUAGE] and in what phase you’ve experienced them. For the symptoms we have already discussed, I may ask you to confirm the phase or phases in which you experience each. [**Dyad Interview Only**: I will also ask that the caregiver speaks up if there any symptoms that are not mentioned by the adolescent].

**To the Caregiver:** Please also mention any symptoms during the [prodrome/alternative language] and [vomiting attack/alternative language] phases.

***[Interviewer to follow instructions and complete Symptom Recording Sheet]***

Symptom Recording Sheet

***[Interviewer: Use this table to record all symptoms as stated by participants. Probe for any symptoms that they have not already mentioned. Record words and terms as used by participants. Note whether they experience the symptom during prodrome and/or emetic phases by marking the appropriate cell with an “X”.]***

| **Symptoms** | **Patient Language/ Terms** | **Experience during Prodromal Phase?** | **Experience during Emetic Phase?** | **Reported as Most Bothersome (X)** | **Spontaneously Reported (S)  or on Probing (P)?** | | **Reported by Patient (P) or Caregiver I or Both (B)** |
| --- | --- | --- | --- | --- | --- | --- | --- |
|  |  |  |  |  | **S** | **P** | **P, C, or B** |
| Nausea, queasiness, feeling sick to stomach |  |  |  |  |  |  |  |
| Vomiting, throwing up |  |  |  |  |  |  |  |
| Retching |  |  |  |  |  |  |  |
| Dry heaves |  |  |  |  |  |  |  |
| Headache |  |  |  |  |  |  |  |
| Migraine |  |  |  |  |  |  |  |
| Belly/abdominal/stomach pain |  |  |  |  |  |  |  |
| Sore stomach muscles |  |  |  |  |  |  |  |
| Skin sensitivity/sensitivity to touch |  |  |  |  |  |  |  |
| Diarrhea |  |  |  |  |  |  |  |
| Pale skin |  |  |  |  |  |  |  |
| Sensitivity to light |  |  |  |  |  |  |  |
| Sensitivity to sound |  |  |  |  |  |  |  |
| Dehydration |  |  |  |  |  |  |  |
| Tiredness/exhaustion/fatigue |  |  |  |  |  |  |  |
| Sweats/hot flashes |  |  |  |  |  |  |  |
| Chills/cold flashes |  |  |  |  |  |  |  |
| Rapid heartbeat |  |  |  |  |  |  |  |
| Dry mouth |  |  |  |  |  |  |  |
| Light headedness |  |  |  |  |  |  |  |
| Anxiety/fear/worry/panic/sense of impending doom |  |  |  |  |  |  |  |
| Other: |  |  |  |  |  |  |  |

1. Of all the symptoms we’ve discussed in either phase, which would you say is the most bothersome to you? Please explain why you chose this one.

**To the Caregiver:**

1. Is there any further information that you wish to add to what your child described?
2. Which symptom is most difficult for you to help your child manage?

#### Triggers, Behavior, and Coping During Emetic Phase

1. Do you notice certain triggers or things that bring on a [vomiting attack/alternate language]? [If yes] Please describe.
2. During a [vomiting attack/alternate language], where in your house or elsewhere do you typically stay while waiting for it to end?
3. Is there a particular room or bathroom that you tend to stay in, or do you move around?
4. Do you fall asleep during a [vomiting attacks/alternate language] or are you awake throughout the entire attack?
5. [If they sleep] Can you tell me how long you tend to sleep or how many times you might fall asleep within a single [vomiting attack/alternate language]?
6. What do you do to get relief from the symptoms during a [vomiting attack/alternate language]? What things do you try to do to help cope with symptoms? For example, some people may take hot showers or baths.
7. Have you ever tried treatment for [CVS/PARTICIPANT LANGUAGE]? [If yes] What was the treatment(s), and did it help?

**To the Caregiver:**

1. [Vomiting attacks/alternate language] can be a difficult time for your child, so recalling details may be hard for them. Is there any additional information that you can provide?
2. Do you have any additional information to provide on your child’s triggers, ways they cope, or what treatments they’ve tried?

Impact of CVS

***[Interviewer: For this portion of the guide, please use the Impact Recording Sheet (below) to document impacts that the participant spontaneously mentions. Do NOT probe on each impact listed in the table]***

Now, let’s talk about how [CVS/PARTICIPANT LANGUAGE] affects your daily life.

#### General Daily Impact

1. How does having [CVS/PARTICIPANT LANGUAGE] impact your everyday life?
2. Probe on sleep, physical activity, daily activities, work and/or school, social activity, friendships, relationships, and emotions.
3. Does your [CVS/PARTICIPANT LANGUAGE] affect any other part of your life that we haven’t discussed yet? [If yes] Please explain.
4. Of all the issues and impacts on your life that we’ve discussed, which one would you say is the **most bothersome** [probe with impacts mentioned, if needed]? Why?

**To the Caregiver:**

1. Do you have any additional information, or would you like to provide examples of other impacts experienced by your child?
2. What impact is most difficult for you and your family?

#### Impacts by Phase

1. Would you say that [CVS/PARTICIPANT LANGUAGE] impacts your life when you’re feeling well and in between episodes?
2. How is your life impacted when you start to sense a [CVS/PARTICIPANT LANGUAGE] episode is coming on (i.e., impacts during a prodrome/alternative language phase)?
3. How is your life impacted while you’re experiencing the [vomiting attack/alternate language] of an episode (i.e., vomiting phase)?
4. How are you impacted after the [vomiting attack/alternate language] when you’re recovering from the episode?
5. How long does it typically take for you to recover from an episode and get back to your “normal self”?
6. How do you know you are fully recovered?

**To the Caregiver:**

1. Is there any further information that you want to add based on what you have witnessed your child experience?
2. How do each of these phases impact you and other members of your household?
3. How do you know your child has fully recovered from their [vomiting attacks/alternate language]?

Impact Recording Sheet

***[Interviewer: Use this table to record impacts spontaneously reported by participants. Do not probe on impacts that are not spontaneously mentioned. Make note of words used by participants. Mark the box with an “X” for any relevant impact discussed by the participant.]***

| Impact | Impact Description | List Most Bothersome Impact | Adolescent Participant’s Language/Notes | Caregiver Participant’s Language/Notes |
| --- | --- | --- | --- | --- |
| 🞎 Sleep (generally) | 🞎 Ability to fall asleep  🞎 Ability to stay asleep  🞎 Ability to fall back to sleep  🞎 Wake up feeling rested |  |  |  |
| 🞎 Physical impacts (generally) | 🞎 Ability to exercise  🞎 Ability to walk or climb stairs  🞎 Needing to rest  🞎 Other |  |  |  |
| 🞎 Daily activities (generally) | 🞎 Hobbies  🞎 Daily routine/planning  🞎 Self-care practices  🞎 Other |  |  |  |
| 🞎 Work or school (generally) | 🞎 Daily work/school routine  🞎 Engage in work/school-related activities  🞎 Work/school absenteeism  🞎 Productivity while at work |  |  |  |
| 🞎 Emotions or mood (generally) | 🞎 Depression  🞎 Anxiety  🞎 Panic  🞎 Irritability  🞎 Embarrassment  🞎 Worry  🞎 Stress  🞎 Frustration  🞎 Other |  |  |  |
| 🞎 Social life (generally) | 🞎 Social activities (e.g. going out -cinema, dinner)  🞎 Leisure activities/hobbies  🞎 Sports (e.g. swimming)  🞎 Travel  🞎 Other |  |  |  |
| 🞎 Relationships (generally) | 🞎 Family relationships  🞎 Friend relationships  🞎 Work/school relationships  🞎 Other |  |  |  |
| 🞎 Other: specify |  |  |  |  |

Wrap-up/Conclusions

Thank you so much for your time today. The information that you have shared will help us better understand the experiences of people living with [CVS/PARTICIPANT LANGUAGE] and help us develop a new questionnaire to measure your [CVS/PARTICIPANT LANGUAGE] symptoms. Your input is extremely helpful.

**END INTERVIEW HERE**

#

# Patient Caregiver Only Interview: Semi-structured Interview Guide

**Participant ID: ___________ Verification Code: _______ Date (MM/DD/YYYY): ____/____/____**

**Instructions for the Interviewer:**

**The purpose of this interview is to capture the CVS experience of the adolescent patient through the opinion of their adult caregiver. Based on the stated preference of the adolescent patient, interviews using this guide should be conducted with the adolescent’s adult caregiver only; a separate one-on-one interview with the respective adolescent should precede this interview.**

**This discussion guide is meant to help focus the discussion but should not be used as a verbatim script; probes and questions may change slightly depending on participant feedback. Additional unscripted probes have been included and should be used to gain further information or clarification. These may include the following:**

Clarification: I don’t quite understand that.

Expressing understanding: How did you cope with that?

Justification: What makes you say that?

Importance: I’m not sure how these two things are linked. Can you please clarify?

Extending narrative: Tell me a bit more about that.

Accuracy: Let’s see if I’ve got that right.

**The interviewer will introduce themselves and explain the study background and interview process. The interview session will be approximately 60-75 minutes of speaking and writing. The interviewer may adapt the guide to cover the topics in the amount of time allotted for the session.**

Introduction

Background

Good [**morning/afternoon/evening**]. I am [**interviewer name**], a research professional working with Evidera, a group focusing on patient-centered research. As part of this study, Evidera is planning to interview about 12 adolescent patients who have been diagnosed with cyclic vomiting syndrome, or CVS, along with their adult caregiver. The study is sponsored by Takeda, a pharmaceutical company, who is developing a treatment for symptoms related to CVS.

Thank you for taking the time to participate in this interview. Based on the preference stated while scheduling the interviews, this interview will be conducted as a one-on-one interview with the adolescent patient’s adult caregiver only; a separate one-on-one interview will be conducted with your adolescent. The information that you share with us today will help us better understand the experience of people living with CVS and help us develop a new questionnaire to measure CVS symptoms. Your input is extremely helpful.

The focus of our discussion today will be on the symptoms related to your adolescent’s CVS, including the impact of these symptoms on their day-to-day life.

As mentioned in the consent form, we would like to audio-record today’s interview so that I can capture everything that you say and review it later. A word-for-word transcript or written documentation of the audio-recording will be prepared. Your privacy will be protected, and the transcript will not include any mention of your name, your adolescent’s name, or information that could be used to discover your identity. You will be identified only with an identification (ID) number to protect your confidentiality.

Is it okay for me to record the conversation today?

**If yes, continue.**

**If no, state the following**: That’s alright. I will be taking detailed notes during the interview, and I may ask you to repeat certain responses, so I capture your information accurately.

Discussion Rules

Before we begin, I’d like to review a few points that will make our discussion more productive.

- There are no right or wrong answers because everyone's experiences are different. Please feel free to share your point of view about how CVS affects your adolescent.
- My role here is to ask questions and to listen. I’ll also be summarizing things you’ve said at times to make sure I understand correctly. I’ll ask questions to obtain your feedback, and I’ll move the discussion from one question to the next, to try to keep us on track so we can finish within the allotted time. Some of my questions might seem repetitive or seem like they have obvious answers, but please bear with me and feel free to ask questions or seek clarification at any time.
- Please talk clearly and speak up as I don’t want to miss any of your comments and feedback.
- We’ll be talking together on a first-name basis, but later in the report, no names will be included or attached to any of your comments.
- I am not a medical doctor, so I am not qualified to give out medical advice, and I may ask you to clarify anything with which I am not familiar.

**Do you have any questions before we begin?**

***Note: Read statement below only if participant(s) gave permission to audio-record the interview.***

**Begin Recorder**: This is participant ID **[insert Participant ID number here]**, and today is **[date]**. I want to confirm that you have reviewed and signed the consent form, that you agree to participate in this study, and that you agree to today’s interview being audio-recorded. **Is that correct?**

Concept Elicitation

So far, I have referred to cyclic vomiting syndrome as CVS. Before we get to the interview questions, I’d like to know from you if there is another name you or your adolescent would prefer to call your adolescent’s CVS?

1. Is there another name for CVS that you would prefer to use?

Experience with Cyclic Vomiting Syndrome

#### Diagnosis History and General Experience of CVS

First, I will ask a few questions about your adolescent’s experiences with cyclic vomiting syndrome. I’ll refer to cyclic vomiting syndrome as [CVS/PARTICIPANT LANGUAGE] moving forward.

1. Do you remember when your adolescent was first told by a doctor that they have [CVS/PARTICIPANT LANGUAGE]?
2. Do you recall what issues or symptoms led you and your adolescent to go to the doctor?
3. Was your adolescent told that they had other issues or medical conditions before being told that they have [CVS/PARTICIPANT LANGUAGE]? Was it difficult to get the correct diagnosis?

#### Experience of CVS Phases in General

As you may know, people experience different phases (or certain periods of time) with their [CVS/PARTICIPANT LANGUAGE] where sometimes they feel well and other times where they don’t feel well.

1. Is that true for your adolescent with [CVS/PARTICIPANT LANGUAGE]?
2. [If yes] Can you describe the different phases they go through and how they feel during each phase?
3. How long does each phase usually last for them?
4. What do you call each time period between [CVS/PARTICIPANT LANGUAGE] episodes before your adolescent begins to feel a new [CVS/PARTICIPANT LANGUAGE] episode coming on?
5. [If no] Can you describe how they are feeling overall during a [CVS/PARTICIPANT LANGUAGE] episode?

#### Experience of Prodrome Phase

Some people with [CVS/PARTICIPANT LANGUAGE] have what is a called a prodrome phase that comes right before a vomiting attack begins. The prodrome phase is where they may feel sick and feel a vomiting attack is coming. This phase can last for minutes or hours.

1. What do you and your adolescent call this phase?
2. Does your adolescent experience a [prodrome/alternative language] phase before they start to vomit?
3. If yes, can you tell when your adolescent is starting a [prodrome/alternative language] phase?
4. What does a typical [prodrome/alternative language] phase look like for them?
5. What [prodrome/alternative language] symptoms do they have? [Interviewer to record symptoms on the Symptom Recording Sheet in the **prodrome column**]
6. Do their [prodrome/alternative language] symptoms change with each [prodrome/alternative language] phase that they experience or are they about the same each time? Please explain.
7. If probes are needed, ask about severity, frequency, and duration of prodrome symptoms.
8. How long do these symptoms usually last before the vomiting starts?
9. Do they ever have [prodrome/alternative language] symptoms but then do NOT go on to have a vomiting attack?
10. [If yes] How often does that happen for them?
11. [If yes] Does this happen on its own (i.e., naturally) or does your adolescent take medications to prevent the vomiting?

#### Experience of Emetic Phase

1. Can you describe what a typical vomiting attack is like for your adolescent? What do you and your adolescent call this phase?
2. What symptoms do they experience during a [vomiting attack/alternate language]? [Interviewer to record symptoms on the Symptom Recording Sheet in the **emesis column**]
3. Are these symptoms the same each time they have a [vomiting attack/alternate language] or do they have different symptoms with each [vomiting attack/alternate language]? Please explain.
4. How long does a typical [vomiting attack/alternate language] last for them?
5. In your mind, is there a difference between retching and vomiting? [If no or caregiver doesn’t know the term, explain that retching is usually defined as a movement or sound of vomiting but without bringing up any contents, liquid, or solids] How do you prefer to quantify them? [Probe: frequency of vomiting/retching movements, or length of time, or other?]
6. Do you consider dry heaves to be the same as retching? [If different] Please explain.
7. On average, how many times do you think your adolescent vomits (or retches [if considered same as vomiting]) in a typical [vomiting attack/alternate language]?
8. Do their [vomiting attacks/alternate language] change (e.g., in how bad, how often, or for how long they last)? In other words, are some [vomiting attacks/alternate language] worse/more frequent/longer than others or are they the same?

#### Frequency and Timing of Episodes with Vomiting

1. How do you and your adolescent define the beginning and end of a CVS episode, which can include more than one phase? What typically happens to signal the end of a CVS episode for your adolescent?
2. How many [CVS/PARTICIPANT LANGUAGE] episodes do you think your adolescent has had over the past year*?* ***[Interviewer, if necessary, clarify that a CVS episode is being discussed using participant’s own language or the following text: When I say episode, I am talking about from when they start to not feel well to being sick until they start to feel well again]****.*
3. Is there a certain time of year or season when they tend to experience more episodes compared with others (e.g., during the school year or during the summer)?
4. [If yes] Please explain.
5. In this time of year or season when they tend to experience more episodes, how often do they occur? [Interviewer to probe if it is daily, weekly, or less frequently]
6. [If no] How often do they tend to have [CVS/PARTICIPANT LANGUAGE] episodes in a typical month?
7. Do you notice that they experience episodes at certain times of the month?
8. [If yes] Please explain. ***[Interviewer to probe if it is earlier, middle, or later in month and why that may be]***
9. What about certain times of the week? For example, are their episodes more likely to occur on weekdays rather than over the weekend?
10. [If yes] Please explain. ***[Interviewer to probe if it is start, middle, or end in the week and why that may be]***
11. Do they tend to have episodes at a certain time of day? For example, are they more likely to experience an episode at night versus in the morning?
12. [If yes] Please explain.
13. How does the frequency and timing of your adolescent’s [CVS/PARTICIPANT LANGUAGE] impact your adolescent?
14. How does the frequency and timing of your adolescent’s [CVS/PARTICIPANT LANGUAGE] impact you and your family?

#### Symptoms Experienced During Prodrome Phase and Emetic Phase

People living with [CVS/PARTICIPANT LANGUAGE] can experience many different symptoms depending on the person. We’ve already discussed some of the different symptoms your adolescent experiences during different phases, namely during the [prodrome/alternative language] phase and during their [vomiting attack/alternate language]. We want to be sure to record all symptoms that they have ever experienced during each phase.

I’m going to go through a list of symptoms and ask which ones your adolescent has experienced because of their [CVS/PARTICIPANT LANGUAGE] and in what phase they’ve experienced them. For the symptoms we have already discussed, I may ask you to confirm the phase or phases in which they experience each.

***[Interviewer to follow instructions and complete Symptom Recording Sheet]***

Symptom Recording Sheet

***[Interviewer: Use this table to record all symptoms as stated by participants. Probe for any symptoms that they have not already mentioned. Record words and terms as used by caregiver participants. Ask caregiver participant if term used by them is the same or different from the term used by their adolescent; if different, note term used by adolescent. If same term, note “same”. If caregiver does not know what term is used by their adolescent, note “Unknown”. Note whether the adolescent experiences the symptom during prodrome and/or emetic phases by marking the appropriate cell with an “X”.]***

| **Symptoms** | **Adult Caregiver Participant Language/ Terms** | **Adolescent Language/ Terms (Note: New Term Name, Same, or Unknown)** | **Experience during Prodromal Phase?** | **Experience during Emetic Phase?** | **Reported as Most Bothersome (X)** | **Spontaneously Reported (S)  or on Probing (P)?** | |
| --- | --- | --- | --- | --- | --- | --- | --- |
|  |  |  |  |  |  | **S** | **P** |
| Nausea, queasiness, feeling sick to one’s stomach |  |  |  |  |  |  |  |
| Vomiting, throwing up |  |  |  |  |  |  |  |
| Retching |  |  |  |  |  |  |  |
| Dry heaves |  |  |  |  |  |  |  |
| Headache |  |  |  |  |  |  |  |
| Migraine |  |  |  |  |  |  |  |
| Belly/abdominal/stomach pain |  |  |  |  |  |  |  |
| Sore stomach muscles |  |  |  |  |  |  |  |
| Skin sensitivity/sensitivity to touch |  |  |  |  |  |  |  |
| Diarrhea |  |  |  |  |  |  |  |
| Pale skin |  |  |  |  |  |  |  |
| Sensitivity to light |  |  |  |  |  |  |  |
| Sensitivity to sound |  |  |  |  |  |  |  |
| Dehydration |  |  |  |  |  |  |  |
| Tiredness/exhaustion/fatigue |  |  |  |  |  |  |  |
| Sweats/hot flashes |  |  |  |  |  |  |  |
| Chills/cold flashes |  |  |  |  |  |  |  |
| Rapid heartbeat |  |  |  |  |  |  |  |
| Dry mouth |  |  |  |  |  |  |  |
| Light headedness |  |  |  |  |  |  |  |
| Anxiety/fear/worry/panic/sense of impending doom |  |  |  |  |  |  |  |
| Other: |  |  |  |  |  |  |  |

Of all the symptoms we’ve discussed in either phase, which would you say is the most bothersome for your adolescent? Is this based on your observation or communication from your adolescent? Please explain why you chose this symptom.

1. Which symptom is the most difficult for you to help your adolescent manage? Please describe why.

#### Triggers, Behavior, and Coping During Emetic Phase

1. Are there certain things that can trigger or bring on a [vomiting attack/alternate language] for your adolescent? [If yes] Please describe.
2. During a [vomiting attack/alternate language], where in your house or elsewhere does your adolescent typically stay while waiting for it to end?
3. Is there a particular room or bathroom that they tend to stay in, or do they move around?
4. Do they fall asleep during a [vomiting attack/alternate language] or are they always awake throughout the entire attack?
5. [If they sleep] Can you tell me how long they tend to sleep for or how many times they might fall asleep within a single [vomiting attack/alternate language]?
6. What does your adolescent (or you) try to do in order to get some relief from symptoms during a [vomiting attack/alternate language]? For example, some people may take hot showers or baths.
7. Has your adolescent ever tried treatment for [CVS/PARTICIPANT LANGUAGE]? [If yes] What was the treatment(s), and did it help?

Impact of CVS

***[Interviewer: For this portion of the guide, please use the Impact Recording Sheet (below) to document impacts that the participant spontaneously mentions. Do NOT probe on each impact listed in the table.]*** Now, let’s talk about how [CVS/PARTICIPANT LANGUAGE] affects your adolescent’s and your daily life.

#### General Daily Impact

1. How does having [CVS/PARTICIPANT LANGUAGE] impact your adolescent’s everyday life?
2. Probe on sleep, physical activity, daily activities, work and/or school, social activity, friendships, relationships, and emotions.
3. Does their [CVS/PARTICIPANT LANGUAGE] affect any other part of their life that we haven’t discussed yet? [If yes] Please explain.
4. Of all the issues and impacts on their life that we’ve discussed, which one would you say is the most bothersome for them [probe with impacts mentioned, if needed]? Why?
5. What impact is most bothersome for you and your family?

#### Impacts by Phase

1. Would you say that [CVS/PARTICIPANT LANGUAGE] impacts the life of your adolescent when they are feeling well and in between episodes?
2. How is their life impacted when they sense a [CVS/PARTICIPANT LANGUAGE] episode is coming on (i.e., impacts during a prodrome/alternative language phase)?
3. How is their life impacted while they’re experiencing the [vomiting attack/alternate language] of an episode (i.e., emetic phase)?
4. How is their life impacted after the [vomiting attack/alternate language] when you’re recovering from the episode?
5. How long does it typically take for them to recover from an episode and to get back to their “normal self”?
6. How do you know your child has fully recovered from their [vomiting attack/alternate language]?
7. How do each of these phases impact you and other members of your household?

Impact Recording Sheet

***[Interviewer: Use this table to record impacts spontaneously reported by participants. Do not probe on impacts that are not spontaneously mentioned. Make note of words used by participants. Mark the box with an “X” for any relevant impact discussed by the participant.]***

| Impact | Impact Description | List Most Bothersome Impact | Caregiver Participant’s Language/Notes |
| --- | --- | --- | --- |
| 🞎 Sleep (generally) | 🞎 Ability to fall asleep  🞎 Ability to stay asleep  🞎 Ability to fall back to sleep  🞎 Wake up feeling rested |  |  |
| 🞎 Physical impacts (generally) | 🞎 Ability to exercise  🞎 Ability to walk or climb stairs  🞎 Needing to rest  🞎 Other |  |  |
| 🞎 Daily activities (generally) | 🞎 Hobbies  🞎 Daily routine/planning  🞎 Self-care practices  🞎 Other |  |  |
| 🞎 Work or school (generally) | 🞎 Daily work/school routine  🞎 Engage in work/school-related activities  🞎 Work/school absenteeism  🞎 Productivity while at work |  |  |
| 🞎 Emotions or mood (generally) | 🞎 Depression  🞎 Anxiety  🞎 Panic  🞎 Irritability  🞎 Embarrassment  🞎 Worry  🞎 Stress  🞎 Frustration  🞎 Other |  |  |
| 🞎 Social life (generally) | 🞎 Social activities (e.g., going out -cinema, dinner)  🞎 Leisure activities/hobbies  🞎 Sports (e.g., swimming)  🞎 Travel  🞎 Other |  |  |
| 🞎 Relationships (generally) | 🞎 Family relationships  🞎 Friend relationships  🞎 Work/school relationships  🞎 Other |  |  |
| 🞎 Other: specify |  |  |  |

Wrap-up/Conclusions

Thank you so much for your time today. The information that you have shared will help us better understand the experiences of people living with [CVS/PARTICIPANT LANGUAGE] and help us develop a new questionnaire to measure [CVS/PARTICIPANT LANGUAGE] symptoms. Your input is extremely helpful.

**END INTERVIEW HERE**
