# Supplementary material for: Understanding the adult and adolescent patient experience with cyclic vomiting syndrome: a concept elicitation study
Source: BMC Gastroenterol. 2025 Feb 17;25:85. doi: 10.1186/s12876-025-03595-7 (PMC11834555; doi:10.1186/s12876-025-03595-7)
Supplement: Supplementary file 2 — Additional file 2. Supplementary tables. Tables on caregiver sociodemographic characteristics, CVS concept elicitation interview quotes, saturation of symptom concepts in the prodrome phase, saturation of symptom concepts in the emetic phase, and CVS impacts. [file 12876_2025_3595_MOESM2_ESM.docx]

# Supplementary Table 1 Caregiver sociodemographic characteristics

| Characteristic | Overall  (*n* = 15) |
| --- | --- |
| Relationship to child (patient), *n* (%) |  |
| Parent | 15 (100) |
| Age (years) |  |
| Mean (SD) | 47.0 (6.7) |
| Median [min, max] | 45.0 [38.0, 59.0] |
| Sex, *n* (%) |  |
| Male | 2 (13.3) |
| Female | 13 (86.7) |
| Ethnic background, *n* (%) |  |
| Not Hispanic or Latino | 15 (100) |
| Racial background, *n* (%) |  |
| White | 14 (93.3) |
| Asian | 1 (6.7) |
| Current living/domestic situation, *n* (%) |  |
| Living with a partner or spouse, family, or friends | 15 (100) |
| Current marital status, *n* (%) |  |
| Married or living in marriage-like relationship | 15 (100) |
| Education status, *n* (%) |  |
| Secondary/high school | 1 (6.7) |
| Some college | 1 (6.7) |
| College degree | 9 (60.0) |
| Postgraduate degree | 3 (20.0) |
| Technical or vocational degree | 1 (6.7) |
| Employment status, *n* (%) |  |
| Work full-time | 7 (46.7) |
| Work part-time | 4 (26.7) |
| Full-time homemaker | 4 (26.7) |
| Annual household income, *n* (%) |  |
| $45,000–$75,000 | 3 (20.0) |
| > $75,000 | 12 (80.0) |

*max* maximum, *min* minimum, *SD* standard deviation

# Supplementary Table 2 CVS concept elicitation interview quotes by topic

| Topic | Adults | Adolescents |
| --- | --- | --- |
| Triggers for CVS episodes | ADU12: “There’s food triggers. Like I’ve had to cut out bell peppers, chili peppers, onions, all that stuff.”  ADU16: “I know good stress and bad stress, which is ironic because I’m in the most stressful kind of job ever. … I don’t really have food triggers but there definitely are smells and environmental triggers that I’ve identified.” | ADO06: “Yeah, traveling is a big one, like flying and like cruises. Those will always put me in an episode, no matter if it was stressful or anything.”  CG13: “Major holidays was always a trigger. … Just the excitement or the anticipation or whatever was linked with that would put her into an episode.” |
| Frequency and timing of CVS episodes | ADU04: “Yeah, more in the wintertime. When it first starts getting cold, November, December, I notice that it gets really bad. And then also once it starts getting very, very hot outside, like the end of June, early July, I get sick a lot as well.”  ADU11: “I notice a trend. I don’t know if it’s going to be like this, this year, but it seemed to be Monday. After the weekend I would start to feel sick. I would start to feel nauseous and then I would start throwing up Monday.” | ADO09: “For me, they more than likely occur on, like, in the beginning of the week, just because like I always, because of like the stress I put on for like school-wise. So, like I had like a good weekend, and then like I’m trying to get like back into that like school flow.”  ADO12: “At least three times more often [during the school year than during the summer].” |
| Symptoms during the prodrome phase | ADU10: “It’s really scary for me. I guess the part about the syncope kind of sets in where my vision and hearing and the sweating because you feel like you’re dying… I feel very weak and like I’m going to faint and sometimes I do. … And sometimes I totally go unconscious and sometimes I don’t.”  ADU11: “I have a time before I get sick where I will get nauseous and hot. Sometimes, occasionally I will get a migraine in my left upper area, like in around my eye. It’ll be like anywhere from a half an hour to maybe even 2 hours...” | ADO11: “I would say the first phase I start burping, I feel it coming on, my mouth gets all dry and doesn’t taste well…”  CG11: “I kind of feel like he’s pretty irritable, but who wouldn’t be at that time, right? But I kind of feel he wants it to be quiet, even though maybe you might not feel like you’re sensitive to sound, it’s just what’s going on might be affecting it too.” |
| Symptoms during the emetic phase | ADU09: “I’ll vomit almost every 15 minutes for hours. Hours and hours and hours. And I can’t drink anything. … I can’t do anything but lay on the floor or lay with my face in a bucket or a trashcan or a bag, whatever is nearby, until it stops. Even in the vomiting phase I still experience the headaches, I still have visual disturbances, and I still have the abdominal pain. And that’s probably the worst because your whole body is… It’s almost like it’s a very violent kind of vomiting.”  ADU12: “Usually, the frequency of that vomiting is anywhere from 5 minutes apart to 30 minutes apart. There’s extreme abdominal cramping. … Now, if I let that go, it will get to the point where I get so dehydrated that we’ve been forced to call emergency services before because I just can’t move.” | ADO06: “I’m so out of it at that point that I honestly don’t know. Like, I’m very nauseous, and that’s really it, that I can feel, I guess. Like I’m not aware of anything.”  CG14: “So he’ll have dry heaves, like really intense headache. He describes it as oh – his stomach, he says he could feel the vomit coming up, but like his headache after he is done vomiting, he has described it like when he was younger, he would say it feels like a freight train is coming through my head or I think I’m getting hit in the head with a hammer, stuff like that. Now he just, when he’s having an intense headache, he’ll like scream from the pain.” |
| Comparison of vomiting vs. retching | ADU11: “Yes. I feel like retching, or what I think it is, is more of like a not getting barely anything up. When you might have a little bit of fluid in your stomach, or your stomach is completely empty, and you’re kind of gagging almost. And then the vomiting is the actual where it comes out, like where you get some up.”  ADU12: “For me, vomiting would be the ability to actually expunge material. Whereas the retching feels just like your muscles are tearing themselves apart.” | ADO05: “I think there is a very distinct difference. I think—yeah, when I retch, that’s when I’m gagging but I do not throw up.”  ADO11: “I would count them as the same thing, to be honest, because, I mean, I would say, if I had something to throw up or to vomit, I would be vomiting it instead of dry heaving.” |
| CVS impacts on daily life | ADU03: “I can’t work, can’t eat. I’m the main provider for me and my wife so that’s stress over maintaining my employment. It’s a big one. Even though I seemingly have it a bit more control now, the times where it was worse historically, aren’t too far from my memory.”  ADU11: “It’s hard. I can drive myself around and do things like that as long as I have my bad and I’m okay. I don’t know how long it would last. I think the last episode that I had that was 2 weeks, it took me like a week to get back on my feet. Eating what I wanted and being able to go and do what I wanted without being nervous about it as much as normal.” | ADO07: “For school, it’s hard to get back on track. It’s hard to turn in a lot of missing work. It’s hard for my grades. It’s just a lot of work. After time, more work builds up, and then it causes more stress, and that causes more headaches, more nausea, so the friend stuff is hard, because I have to cancel a lot of things, and I might disappoint them.”  ADO11: “Missing out on sports and family events, because those are the two things that are like what I do for most of the time, other than school, and it really sucks that I have to miss those.” |

# *ADO* adolescent, *ADU* adult, *CG* caregiver, *CVS* cyclic vomiting syndrome

# Supplementary Table 3 Saturation of symptom concepts in the adult and adolescent patient populations: prodrome phase

| Symptom, *n* (%) | Adults | | | | Adolescents | | | |
| --- | --- | --- | --- | --- | --- | --- | --- | --- |
|  | Total endorsing symptom  (*n* = 13) | First transcript  group  (Int. 1–5) | Second transcript  group  (Int. 6–9) | Third transcript  group  (Int. 10–13) | Total endorsing symptom  (*n* = 15) | First transcript  group  (Int. 1–5) | Second transcript  group  (Int. 6–9) | Third transcript  group  (Int. 10–15) |
| Nausea, queasiness, feeling sick to one’s stomach | 12 (92.3) | ✓ | ✓ | ✓ | 15 (100) | ✓ | ✓ | ✓ |
| Anxiety/fear/worry/panic/ sense of impending doom | 10 (76.9) | ✓ | ✓ | ✓ | 9 (60.0) | ✓ | ✓ | ✓ |
| Belly/abdominal/stomach pain | 9 (69.2) | ✓ | ✓ | ✓ | 11 (73.3) | ✓ | ✓ | ✓ |
| Tiredness/exhaustion/fatigue | 7 (53.8) | ✓ | ✓ | ✓ | 9 (60.0) | ✓ | ✓ | ✓ |
| Sensitivity to light | 7 (53.8) | ✓ | ✓ | ✓ | 7 (46.7) |  | ✓ | ✓ |
| Dry mouth | 6 (46.2) | ✓ | ✓ |  | 1 (6.7) |  |  | ✓ |
| Lightheadedness | 6 (46.2) | ✓ | ✓ | ✓ | 10 (66.7) | ✓ | ✓ | ✓ |
| Rapid heartbeat | 5 (38.5) | ✓ | ✓ | ✓ | 2 (13.3) | ✓ | ✓ |  |
| Sweats/hot flashes | 5 (38.5) | ✓ | ✓ | ✓ | 9 (60.0) | ✓ | ✓ | ✓ |
| Sensitivity to sound | 5 (38.5) | ✓ | ✓ | ✓ | 5 (33.3) |  | ✓ | ✓ |
| Headache | 4 (30.8) | ✓ | ✓ | ✓ | 11 (73.3) | ✓ | ✓ | ✓ |
| Chills/cold flashes | 4 (30.8) | ✓ | ✓ | ✓ | 7 (46.7) | ✓ | ✓ | ✓ |
| Pale skin | 4 (30.8) | ✓ | ✓ | ✓ | 11 (73.3) | ✓ | ✓ | ✓ |
| Dehydration | 3 (23.1) | ✓ | ✓ |  | 3 (20.0) | ✓ | ✓ | ✓ |
| Migraine | 3 (23.1) | ✓ |  | ✓ | 8 (53.3) | ✓ | ✓ | ✓ |
| Diarrhea | 2 (15.4) |  | ✓ | ✓ | 4 (26.7) | ✓ | ✓ | ✓ |
| Skin sensitivity/sensitivity  to touch | 1 (7.7) |  |  | ✓ | 3 (20.0) | ✓ | ✓ | ✓ |
| Retching | 1 (7.7) |  |  | ✓ |  |  |  |  |
| Sore stomach muscles |  |  |  |  | 1 (6.7) |  | ✓ |  |
| Spontaneously reported symptoms^a^ | 12 (92.3) | ✓ | ✓ | ✓ | 10 (66.7) | ✓ | ✓ | ✓ |
| Fainting/syncope | 2 (15.4) | ✓ |  |  |  |  |  |  |
| Thirsty | 2 (15.4) | ✓ |  | ✓ |  |  |  |  |
| Fullness in belly/blocked stomach | 2 (15.4) | ✓ | ✓ |  |  |  |  |  |
| Irritability | 2 (15.4) | ✓ | ✓ |  | 2 (13.3) |  |  | ✓ |
| Loss of appetite | 2 (15.4) |  | ✓ |  |  |  |  |  |
| Salivation | 2 (15.4) |  | ✓ | ✓ |  |  |  |  |
| Brain fog | 1 (7.7) | ✓ |  |  |  |  |  |  |
| Hearing/vision muffled – sees stars | 1 (7.7) | ✓ |  |  |  |  |  |  |
| Weakness | 1 (7.7) | ✓ |  |  |  |  |  |  |
| Eye pupils dilate | 1 (7.7) | ✓ |  |  |  |  |  |  |
| Blurred vision | 1 (7.7) | ✓ |  |  |  |  |  |  |
| Gas (burping/bloating/flatulence) flatulence flatulence | 1 (7.7) |  | ✓ |  | 2 (13.3) |  |  | ✓ |
| Red flushing on nose and cheeks | 1 (7.7) |  | ✓ |  |  |  |  |  |
| Reflux | 1 (7.7) |  | ✓ |  |  |  |  |  |
| Back pain | 1 (7.7) |  | ✓ |  |  |  |  |  |
| Malaise | 1 (7.7) |  | ✓ |  |  |  |  |  |
| Sensitivity to smells | 1 (7.7) |  |  | ✓ | 1 (6.7) | ✓ |  |  |
| Muscle aches |  |  |  |  | 2 (13.3) | ✓ |  |  |
| Fever (low-grade) |  |  |  |  | 1 (6.7) | ✓ |  |  |
| Flushing of skin (ears) |  |  |  |  | 1 (6.7) | ✓ |  |  |
| Pressure in head |  |  |  |  | 1 (6.7) | ✓ |  |  |
| Body shudders |  |  |  |  | 1 (6.7) |  | ✓ |  |
| Dark circles under eyes |  |  |  |  | 1 (6.7) |  | ✓ |  |
| Mouth waters |  |  |  |  | 1 (6.7) |  | ✓ |  |
| “Out of it”/conscious coma |  |  |  |  | 1 (6.7) |  | ✓ |  |
| Unsettled stomach/rumbling stomach |  |  |  |  | 1 (6.7) |  | ✓ |  |
| Chest pain |  |  |  |  | 1 (6.7) |  |  | ✓ |
| Shakiness |  |  |  |  | 1 (6.7) |  |  | ✓ |

*Int* interview

Green cells indicate the first transcript group in which the symptom was mentioned by a participant

^a^Symptoms were reported by patients and were not probed on by interviewers

# Supplementary Table 4 Saturation of symptom concepts in the adult patient population: emetic phase

| Symptom | Adults | | | | Adolescents | | | |
| --- | --- | --- | --- | --- | --- | --- | --- | --- |
|  | Total endorsing symptom  (*n* = 13) | First transcript  group  (Int. 1–5) | Second transcript  group  (Int. 6–9) | Third transcript  group  (Int. 10–13) | Total endorsing symptom  (*n* = 15) | First transcript  group  (Int. 1–5) | Second transcript  group  (Int. 6–9) | Third transcript  group  (Int. 10–15) |
| Nausea, queasiness, feeling sick to one’s stomach | 13 (100) | ✓ | ✓ | ✓ | 15 (100) | ✓ | ✓ | ✓ |
| Tiredness/exhaustion/ fatigue | 13 (100) | ✓ | ✓ | ✓ | 13 (86.7) | ✓ | ✓ | ✓ |
| Dry heaves | 13 (100) | ✓ | ✓ | ✓ | 12 (80.0) | ✓ | ✓ | ✓ |
| Vomiting, throwing up | 12 (92.3)^a^ | ✓ | ✓ | ✓ | 15 (100) | ✓ | ✓ | ✓ |
| Retching | 12 (92.3) | ✓ | ✓ | ✓ | 11 (73.3) | ✓ | ✓ | ✓ |
| Dehydration | 11 (84.6) | ✓ | ✓ | ✓ | 13 (86.7) | ✓ | ✓ | ✓ |
| Sweats/hot flashes | 10 (76.9) | ✓ | ✓ | ✓ | 10 (66.7) | ✓ | ✓ | ✓ |
| Lightheadedness | 10 (76.9) | ✓ | ✓ | ✓ | 13 (86.7) | ✓ | ✓ | ✓ |
| Abdominal pain  (sore stomach muscles) | 9 (69.2) | ✓ | ✓ | ✓ | 10 (66.7) | ✓ | ✓ | ✓ |
| Pale skin | 9 (69.2) | ✓ | ✓ | ✓ | 11 (73.3) | ✓ | ✓ | ✓ |
| Belly/abdominal/stomach pain | 7 (53.8) | ✓ | ✓ | ✓ | 14 (93.3) | ✓ | ✓ | ✓ |
| Sensitivity to light | 7 (53.8) | ✓ | ✓ | ✓ | 10 (66.7) | ✓ | ✓ | ✓ |
| Headache | 7 (53.8) | ✓ | ✓ | ✓ | 10 (66.7) | ✓ | ✓ | ✓ |
| Anxiety/fear/worry/panic/ sense of impending doom | 7 (53.8) | ✓ | ✓ | ✓ | 7 (46.7) | ✓ | ✓ | ✓ |
| Rapid heartbeat | 6 (46.2) | ✓ | ✓ | ✓ | 8 (53.3) | ✓ | ✓ | ✓ |
| Diarrhea | 6 (46.2) | ✓ | ✓ | ✓ | 7 (46.7) | ✓ | ✓ | ✓ |
| Chills/cold flashes | 6 (46.2) | ✓ | ✓ | ✓ | 7 (46.7) | ✓ | ✓ | ✓ |
| Sensitivity to sound | 6 (46.2) |  | ✓ | ✓ | 9 (60.0) | ✓ | ✓ | ✓ |
| Migraine | 5 (38.5) | ✓ | ✓ | ✓ | 7 (46.7) | ✓ | ✓ | ✓ |
| Dry mouth | 4 (30.8) |  | ✓ | ✓ | 6 (40.0) | ✓ | ✓ | ✓ |
| Sensitivity to touch | 2 (15.4) |  | ✓ | ✓ | 7 (46.7) | ✓ | ✓ | ✓ |
| Spontaneously reported symptoms^b^ | 9 (69.2) | ✓ | ✓ | ✓ | 10 (66.7) | ✓ | ✓ | ✓ |
| Thirsty | 3 (23.1) | ✓ |  | ✓ |  |  |  |  |
| Fainting/syncope | 2 (15.4) | ✓ | ✓ |  |  |  |  |  |
| Loss of appetite | 2 (15.4) |  | ✓ |  |  |  |  |  |
| Watery mouth | 2 (15.4) |  | ✓ |  | 1 (6.7) |  | ✓ |  |
| Hearing/vision muffled –sees stars | 1 (7.7) | ✓ |  |  |  |  |  |  |
| Numbness/tingling  of limbs | 1 (7.7) | ✓ |  |  |  |  |  |  |
| Brain fog | 1 (7.7) | ✓ |  |  |  |  |  |  |
| Pain in esophagus | 1 (7.7) | ✓ |  |  |  |  |  |  |
| Sore/raw lips | 1 (7.7) | ✓ |  |  |  |  |  |  |
| Blurred vision | 1 (7.7) | ✓ |  |  |  |  |  |  |
| Balance issues (vertigo) | 1 (7.7) | ✓ |  |  |  |  |  |  |
| Red flushing on nose and cheeks | 1 (7.7) |  | ✓ |  |  |  |  |  |
| Shivering/chattering teeth but not chills | 1 (7.7) |  | ✓ |  |  |  |  |  |
| Cloudy urine | 1 (7.7) |  | ✓ |  |  |  |  |  |
| Fullness in belly/ blocked stomach | 1 (7.7) |  | ✓ |  |  |  |  |  |
| Weakness in extremities | 1 (7.7) |  |  | ✓ |  |  |  |  |
| High blood pressure | 1 (7.7) |  |  | ✓ |  |  |  |  |
| Shakiness |  |  |  |  | 2 (13.3) | ✓ |  |  |
| Sore throat |  |  |  |  | 2 (13.3) | ✓ |  |  |
| Hunger |  |  |  |  | 1 (6.7) | ✓ |  |  |
| Low-grade fever |  |  |  |  | 1 (6.7) | ✓ |  |  |
| Chest/throat burning |  |  |  |  | 1 (6.7) |  | ✓ |  |
| Dark circles under eyes |  |  |  |  | 1 (6.7) |  | ✓ |  |
| “Out of it”/conscious coma |  |  |  |  | 1 (6.7) |  | ✓ |  |
| Irritability |  |  |  |  | 1 (6.7) |  |  | ✓ |
| Out of breath |  |  |  |  | 1 (6.7) |  |  | ✓ |
| Tingling – face and feet |  |  |  |  | 1 (6.7%) |  |  | ✓ |

*Int* interview

Green cells indicate the first transcript group in which the symptom was mentioned by an adult, adolescent, and/or caregiver

^a^One participant reported no longer vomiting due to strict adherence to abortive medications

^b^Symptoms were reported by patients and not probed on by interviewers

# Supplementary Table 5 CVS impacts reported by adult and adolescent patients

| Impacts | Adults | | | Adolescents | | |
| --- | --- | --- | --- | --- | --- | --- |
|  | Total  (*n* = 13) | Total  (%) | Total  (*n* = 15) | Total  (%) | Adolescent (*n*) | Caregiver (*n*) |
| Sleep (generally) | 12 | 92.3 | 10 | 66.7 |  |  |
| Ability to fall asleep | 3 | 25.0 | 4 | 40.0 | 4 | 0 |
| Ability to stay asleep | 10 | 83.3 | 9 | 90.0 | 9 | 0 |
| Ability to fall back asleep | 2 | 16.7 | 3 | 30.0 | 3 | 0 |
| Wake up feeling rested | 1 | 8.3 | 5 | 50.0 | 5 | 0 |
| Needing to go to sleep early | 0 | 0 | 2 | 20.0 | 1 | 1 |
| Physical impacts | 12 | 92.3 | 11 | 73.3 |  |  |
| Ability to exercise | 5 | 41.7 | 4 | 36.4 | 4 | 0 |
| Ability to walk or climb stairs | 4 | 33.3 | 2 | 18.2 | 2 | 0 |
| Needing to rest | 3 | 25.0 | 8 | 72.7 | 8 | 0 |
| Daily activities | 11 | 84.6 | 10 | 66.7 |  |  |
| Chores/housework/gardening | 4 | 36.4 | 0 | 0 | 0 | 0 |
| Daily routine/planning | 4 | 36.4 | 6 | 60.0 | 6 | 0 |
| Self-care practices | 0 | 0 | 5 | 50.0 | 5 | 0 |
| Hobbies | 0 | 0 | 4 | 40.0 | 4 | 0 |
| Work or school (generally) | 12 | 92.3 | 15 | 100 |  |  |
| Daily work/school routine | 5 | 41.7 | 9 | 60.0 | 9 | 0 |
| Engage in work/school-related activities | 3 | 25.0 | 8 | 53.3 | 8 | 0 |
| Work/school absenteeism | 5 | 41.7 | 13 | 86.7 | 11 | 2 |
| Productivity while at work | 3 | 25.0 | 3 | 20.0 | 3 | 0 |
| Having to flex days at school | NA | NA | 1 | 6.7 | 1 | 0 |
| Emotions or mood (generally) | 13 | 100 | 15 | 100 |  |  |
| Depression | 4 | 30.8 | 3 | 20.0 | 3 | 0 |
| Anxiety | 9 | 69.2 | 10 | 66.7 | 8 | 2 |
| Panic | 3 | 23.1 | 5 | 33.3 | 5 | 0 |
| Irritability | 1 | 7.7 | 8 | 53.3 | 7 | 1 |
| Embarrassment | 1 | 7.7 | 6 | 40.0 | 5 | 1 |
| Worry | 6 | 46.2 | 7 | 46.7 | 6 | 1 |
| Stress | 3 | 23.1 | 7 | 46.7 | 6 | 1 |
| Frustration | 1 | 7.7 | 8 | 53.3 | 8 | 0 |
| Social life (generally) | 12 | 92.3 | 15 | 100 |  |  |
| Social activities | 9 | 75.0 | 14 | 93.3 | 12 | 2 |
| Leisure activities | 1 | 8.3 | 4 | 26.7 | 3 | 1 |
| Travel | 1 | 8.3 | 5 | 33.3 | 3 | 2 |
| Sports | 0 | 0 | 9 | 60.0 | 8 | 1 |
| Church service | 0 | 0 | 2 | 13.3 | 2 | 0 |
| Shopping | 0 | 0 | 1 | 6.7 | 1 | 0 |
| Relationships (generally) | 11 | 84.6 | 12 | 80.0 |  |  |
| Family relationships | 8 | 72.7 | 5 | 41.7 | 4 | 1 |
| Friend relationships | 3 | 27.3 | 9 | 75.0 | 7 | 2 |

*CVS* cyclic vomiting syndrome, *NA* not applicable
